# Reversal in Solvatochromism, enol-imine/keto-amine Tautomerism and (*E*)-(*Z*) Photoisomerizations in a Benzylidene Aniline Schiff Base Derivative in Different Solvents

**DOI:** 10.3390/molecules30030745

**Published:** 2025-02-06

**Authors:** İsa Sıdır, Yadigar Gülseven Sıdır, Halil Berber, Maria L. Ramos, Licínia L. G. Justino, Rui Fausto

**Affiliations:** 1 Department of Physics, Faculty of Sciences and Letters, Bitlis Eren University, Bitlis 13000, Türkiye; ygsidir@beu.edu.tr; 2 CQC–IMS, Department of Chemistry, University of Coimbra, 3004-535 Coimbra, Portugal; mlramos@ci.uc.pt (M.L.R.); liciniaj@ci.uc.pt (L.L.G.J.); 3 Department of Chemistry, Faculty of Sciences, Eskişehir Technical University, Eskişehir 26470, Türkiye; hlberber@eskisehir.edu.tr; 4 ERA-Chair Spectroscopy@IKU, Faculty of Sciences and Letters, Department of Physics, Istanbul Kultur University, Ataköy Campus, Bakirköy, Istanbul 34156, Türkiye

**Keywords:** Solvatochromism, isomerism, tautomerism, hydrogen bonding, benzylidene aniline Schiff base-type imine dye

## Abstract

A novel Schiff base, (*E*)-4-acetyl-*N*-(4-hydroxy-3-methoxybenzylidene)aniline (abbreviated as A*N*HMA), was synthesized and characterized using infrared and ^1^H- and ^13^C-NMR spectroscopies. Optical properties in different solvents were evaluated using UV-vis absorption spectroscopy. The compound is shown to exhibit both positive and negative solvatochromism with reversal occurring for solvents with *E*_T_(30)~45 (e.g., DMSO). The solvatochromic behavior of the compound was found to be strongly dependent on the hydrogen bond abilities and polarizability of the solvent, the observed reversal in solvatochromism being explained by the change in the dominant solvent effects in non-polar and polar–aprotic solvents (H-bond acceptor ability of the solvent and polarizability) compared to polar–protic solvents (H-bond donor ability), according to the developed Catalán multiparametric solvatochromic model. In all freshly prepared solutions studied, the (*E*)-enol-imine tautomer of the compound was found to strongly predominate over the keto-amine tautomeric forms, the latter increasing their populations over time in the presence of H-bond donor/acceptor species. Irradiation of A*N*HMA with UV light (*λ* ≥ 311 nm) was also investigated in several solvents and shown to follow a general pattern, with the conversion of the (*E*)-enol-imine tautomer into the keto-amine forms in a solvent-mediated enol-imine/keto-amine tautomerism, and (*Z*)→(*E*) C=C isomerization between the keto-imine forms. The experimental results received support from an extensive series of calculations on the structure and UV-vis spectra of the relevant tautomeric/isomeric forms of the compound performed at the DFT(B3LYP)/6-311++G(d,p) level of approximation (including time-dependent DFT calculations and solvent consideration).

## 1. Introduction

Solvent effects play a major role in chemistry, affecting kinetics, equilibria, and the reaction mechanisms and determining solubility and acid–base and redox strengths for example; they also affect physical properties of the solutes as, for instance, spectroscopic responses [1,2,3]. Solute–solvent interactions can be of different types and determined both by the physical and chemical properties of the solvents, such as their hydrogen bond donor/acceptor abilities, polarizability, polarity, and dipolarity. The combination of these types of interactions expresses the overall solvent effect on a given property of the solute as solubility or optical properties [1,2,3,4]. In the latter case, for example, it is well known that the position and/or intensity of the bands observed in the absorption and fluorescence electronic spectra of a dye can be remarkably influenced by solvent properties. In the simplest cases, such solvent-induced changes in the characteristics of the spectra of the dye reflect the perturbation imposed by the solvent to the electronic energy levels of the dye, and result in the variation of the color of the solutions of the same solute in different solvents, or solvatochromism [1,2,3] (The term solvatochromism does not strictly apply to solvent effects on the spectral properties of the solute in the visible region, so that the word “color” is here used lato sensu). To understand the solvatochromic behavior of a molecule, solvatochromic models have been developed, which describe the properties of the solvents in terms of a series of empirically determined parameters [1,2,3,5,6,7,8,9]. In this realm, solvent polarity scales are of fundamental importance, and many solvent polarity scales have been proposed, among which the *E*_T_(30) scale, based on Reichardt’s pyridinium *N*-phenolate betaine dye [1,10,11,12,13,14], is the most used.

The Reichardt’s *E*_T_(30) solvent polarity scale expresses the polarity of a given solvent in terms of the energy of the maximum of the band corresponding to the HOMO → LUMO charge-transfer electronic absorption of the *N*-phenolate betaine dye in that solvent (Equation (1), where *λ*_max_ is the wavelength of maximum of the absorption band of the dye) [1]. Other dyes can be used to assess the polarity of the solvents using a similar approach and an equation similar to Equation (1), but where *E*_T_(30) is replaced by *E*_T_(dye) [5,12,13,14,15].

*E*_T_(30) (kcal mol^−1^) = 28,591.5/(*λ*_max_*,* nm) (1)

Solvatochromic organic dyes are important functional materials for a large number of practical applications [16,17]. They present the advantage over other systems in that their solvatochromic properties can be tuned for a given application by tailoring appropriate π-conjugated electron-acceptor/electron-donor systems through the design of molecules, which bear different substituents in a basic molecular scaffold. By changing judiciously the molecular architecture, a family of dyes may comprehend members displaying negative as well as positive solvatochromism, i.e., exhibiting bathochromic (red) or hypsochromic (blue) shifts in the absorption (or fluorescence) maxima with increasing solvent polarity, respectively. Some species can show a reversal in solvatochromism, where red and blue shifts are observed for the same molecule in different regions of solvent polarity. Reversals in solvatochromism have been described over the past four decades, in particular for imine dyes [13,18,19], and have been explained on the basis of different factors: (i) protonation/deprotonation and solvent-assisted prototropism [11,19,20], (ii) differential intermolecular hydrogen bonding with solvent [21], (iii) changes in molecular conformation [22,23], (iv) self-aggregation [24], and (v) variable solvent polarity effects on electronic ground and excited states of the solute [12,13,18,25].

Schiff bases derived from salicylic aldehydes and benzylidene aniline-type imine dyes have been given special attention in relation to solvatochromism [9,26,27,28,29,30,31]. This is mostly because these types of compounds possess specific structural characteristics that can be easily manipulated to fine-tune their properties. In the case of benzylidene aniline-type imine dyes, both intramolecular proton transfer leading to enol-imine/keto-amine tautomerism and (*E*)-(*Z*) isomerization around the imino group (C=N) have been found to be relevant in determining solvatochromism [32,33,34,35,36,37,38,39], and these processes are well known to be strongly dependent on the specific substituents present in the aromatic rings. On these topics, several comprehensive reviews have been published, including those by Minkin and coworkers [38], Flores-Leonar et al. [40], Rahimova [41], and Brewer and coworkers [42]. Because the relevance of the (*E*)-(*Z*) isomerism, which in these compounds can in general be efficiently trigged by light, benzylidene aniline-type imine dyes have also been proposed as molecular switches [43,44] (mimicking the naturally occurring (*E*)-(*Z*) photoisomerization of retinal [45,46]). Due to the sensitivity of the optical properties of these dyes to the solvents’ characteristics and their influence on the relative importance of the different tautomeric forms of the compound, solvents play a critical role in the photochemistry of benzylidene aniline-type imine dyes and, then, in their possible applications as molecular switches and as photochromic sensors, for example.

In this study, the solvatochromic properties exhibited by a novel benzylidene aniline-type imine dye, the Schiff base derived from 4-acetylaniline and 4-hydroxy-3-methoxybenzaldehyde, (*E*)-4-acetyl-*N*-(4-hydroxy-3-methoxybenzylidene)aniline (abbreviated as A*N*HMA), was investigated in 19 solvents. The compound was synthesized and characterized using infrared and ^1^H- and ^13^C-NMR spectroscopies, and its solvatochromism was investigated in the light of the Catalán multiparametric empirical model [1,6]. As shown below, A*N*HMA was found to show a reversal in solvatochromism for a solvent *E*_T_(30) ~45 kcal mol^−1^, which is a consequence of the different dominant solvent effects in non-polar and polar–aprotic solvents (H-bond acceptor ability of the solvent and polarizability) compared to polar–protic solvents (H-bond donor ability), as shown by the developed Catalán multiparametric solvatochromic model.

Furthermore, (*E*)-(*Z*) photoisomerizations about the C=C bond and enol-imine/keto-amine phototautomerization of A*N*HMA upon UV light (*λ* ≥ 311 nm) irradiation were also investigated in several solvents, using UV-vis spectroscopy complemented by density functional theory (DFT) calculations (including time-dependent DFT (TD-DFT) calculations and solvent consideration), demonstrating the sensitivity of the photochemical reactivity of these dyes to the solvents’ characteristics.

## 2. Results and Discussion

### 2.1. Reversal in Solvatochromism of ANHMA

**Figure 1:**
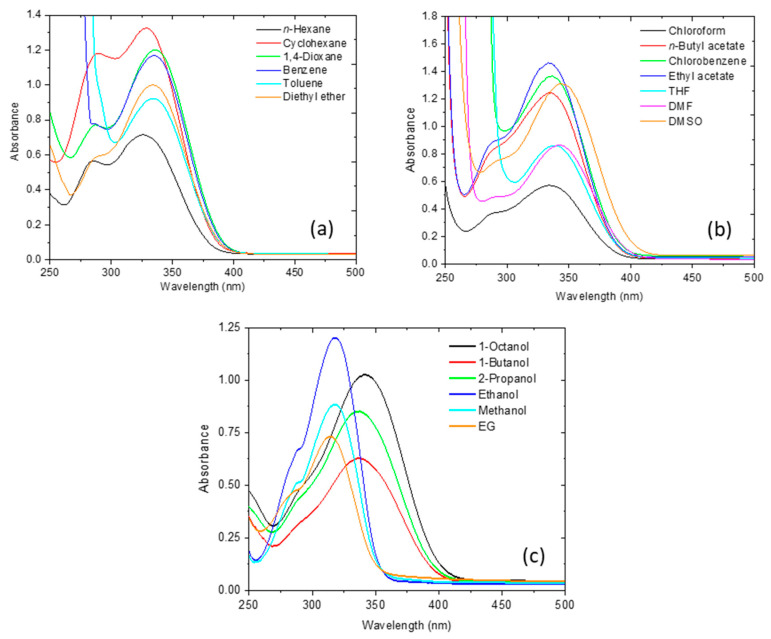
UV-vis absorption spectra of A*N*HMA in non-polar (**a**), polar–aprotic (**b**), and polar–protic (**c**) solvents. THF: Tetrahydrofuran, DMF: *N*,*N*-Dimethylformamide, DMSO: Dimethyl sulfoxide, EG: Ethylene glycol.

The studied compound is a white dust material that gives rise to colorless solutions. Figure 1 shows the room temperature UV-vis absorption spectra of A*N*HMA in solutions of three types of solvents: non-polar, polar–aprotic, and polar–protic. Above 250 nm, the spectrum of the compound exhibits two clearly discernible strong bands, with maxima in the ca. 310–345 nm range (*λ*_max_^II^; most intense band II) and around 285–300 nm (*λ*_max_^I^), in all studied solvents except in the cases of chlorobenzene and tetrahydrofuran (THF) where the shorter wavelength band is overlapped by an intense solvent band (in benzene and in toluene solvent bands also overlap this band extensively, but it is still noticeable).

As discussed in detail in Section 2.3, these bands are ascribed to the (*E*)-enol-imine tautomer of the studied compound, which was found to strongly predominate in all investigated solvents over the keto-amine forms. Table 1 lists the maximum absorption wavelengths of the two bands in the different solvents and the corresponding *E*_T_(A*N*HMA) values.

In the studied non-polar solvents (solvents 1 to 6; see Table 1) *λ*_max_^I^ and *λ*_max_^II^ are observed between 285 and 291 nm and between 326 and 335 nm, respectively, with a trend to exhibit a red shift with the increase of Reichardt *E*_T_(30) solvent parameter that is more noticeable for *λ*_max_^II^, which globally shows a stronger dependence on the solvent.

For the majority of the polar–aprotic solvents investigated (7–13), *λ*_max_^II^ is observed nearly in the same range as in the case of non-polar solvents, 333–339 nm, exhibiting larger red shifts (to 342 and 344 nm) for the two polar–aprotic solvents with the highest *E*_T_(30) values, dimethylformamide (DMF) and dimethylsulfoxide (DMSO), respectively. Taking as reference the shortest wavelength observed in *n*-hexane solution (326 nm), a bathochromic shift of 18 nm in the *λ*_max_^II^ maximum of the most intense A*N*HMA absorption band is observed for DMSO. On the other hand, in all polar–aprotic solvents *λ*_max_^I^ is observed within the range 293–299 nm, which shows a general red shift compared to the region where *λ*_max_^I^ is observed in non-polar solvents (285–291 nm), the longer wavelength being observed in DMSO, following the pattern also seen for *λ*_max_^II^.

In the case of polar–protic solvents (14–19), *λ*_max_^II^ is within the 337–342 nm range for 1-octanol, 2-propanol, and 1-butanol. The solvents in this group have smaller *E*_T_(30), which do not differ much from that observed in non-polar and polar–aprotic solvents, and shift to shorter wavelengths (314–317 nm) in the more polar solvents, ethanol, methanol, and ethylene glycol (EG). A maximum blue shift of 28 nm is observed when comparing the position of *λ*_max_^II^ in EG with that observed in 1-octanol (see Table 1). Though the differences in the *λ*_max_^I^ values are comparatively smaller than those found for *λ*_max_^II^, a similar general trend is observed for the values of *λ*_max_^I^ in polar–protic solvents: it is observed in the 290–292 nm range for the solvents with smaller *E*_T_(30) in this group and within the 287–288 nm interval for those with a higher *E*_T_(30), the maximum blue shift of 5 nm being observed between the position of *λ*_max_^I^ in EG and in 1-octanol.

**Figure 2:**
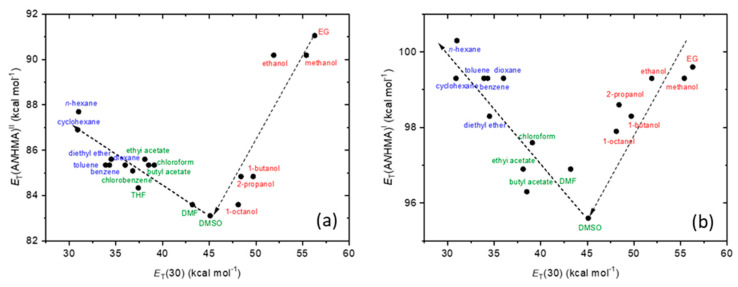
Plot of *E*_T_(A*N*HMA)^II^ (**a**) and *E*_T_(A*N*HMA)^I^ (**b**) values obtained from *λ*_max_^II^ and *λ*_max_^I^ vs. Reichardt’s *E*_T_(30) solvents’ parameter. Color code: blue, non-polar solvents; green, polar–aprotic solvents; red, polar–protic solvents. The arrows shall be taken as guidelines.

The *E*_T_(A*N*HMA) values obtained from the UV absorption spectra in the different solvents are plotted as a function of the solvents’ *E*_T_(30) parameter in Figure 2. This plot highlights the reversal in solvatochromism exhibited by the dye. In going from the non-polar solvent cyclohexane (*E*_T_(30) = 30.9 kcal mol^−1^) to the polar–aprotic solvent DMSO (*E*_T_(30) = 45.1 kcal mol^−1^), the *E*_T_(A*N*HMA)^II^ values decrease from 86.9 to 83.1 kcal mol^−1^ and the *E*_T_(A*N*HMA)^I^ values from ca. 100 to 95.6 kcal mol^−1^ with the increase of the polarity of the solvent (positive solvatochromism). On the other hand, in going from DMSO to the polar–protic solvent with higher *E*_T_(30), EG (*E*_T_(30) = 56.3 kcal mol^−1^), the *E*_T_(A*N*HMA)^II^ and *E*_T_(A*N*HMA)^I^ values increase from 83.1 to 91.1 kcal mol^−1^ and from 95.6 to 99.6 kcal mol^−1^, respectively (negative solvatochromism). Thus, A*N*HMA exhibits a general positive solvatochromism for solvents with *E*_T_(30) values in the ~34–45 kcal mol^−1^ range and negative solvatochromism for solvents with *E*_T_(30) values higher than ~45 kcal mol^−1^, the reversal occurring at an *E*_T_(30) value of *ca*. 45 kcal mol^−1^, which is characteristic of DMSO.

### 2.2. Solvatochromism in ANHMA at the Light of the Catalán Multiparametric Model

The Catalán approach to solvatochromism is based on a multiparametric empirical relation, as given by Equation (2) [1,6]:

*E*_T_(dye) = *E*_T_(dye)_0_ + *a*(SA) + *b*(SB) + *d*(SP) + *e*(SdP) (2)

where *E*_T_(dye)_0_ is a parameter representing the *E*_T_(dye) value for a solvent-unaffected dye, and *a*, *b*, *d*, and *e* are the coefficients that reflect the contribution of the different solvent parameters to the *E*_T_(dye) values determined from the observed band shifts in the different solvents. The parameters SA, SB, SP, and SdP relate to the hydrogen bond donor and hydrogen bond acceptor abilities, polarizability, and dipolarity of the solvent, respectively, and are provided in Table 2 for the relevant solvents [1,6].

Multiple linear regression using Equation (2), applied to the *E*_T_(A*N*HMA)^II^ and *E*_T_(A*N*HMA)^I^ experimental data, yields the following solutions:

*E*_T_(A*N*HMA)^II^ = 96.2(±2.8) + 8.7(±1.3)SA − 6.2(±1.3)SB − 12.9(±3.9)SP + 0.9(±1.3)SdP (3)

*E*_T_(A*N*HMA)^I^ = 101.5(±2.1) + 4.7(±1.0)SA − 1.7(±0.9)SB − 2.1(±2.9)SP − 2.9(±0.9)SdP (4)

where the correlation coefficient and the statistical parameter (*R*^2^, F) equal to (0.848, 19.5) and (0.756, 9.3), respectively.

In both Equations (3) and (4), coefficient *a* is positive, while *b* and *d* are negative; coefficient *e* is positive in the model derived from *E*_T_(A*N*HMA)^II^ (Equation (3), which refers to band II) and negative in the model derived from *E*_T_(A*N*HMA)^I^ (Equation (4)). For band II, the absolute values of the coefficients follow the order *d* > *a* > *b* >> *e*, while for band I these values relate like *a* > *e* ≈ *d* ≈ *b*. It can then be concluded that the changes in the *E*_T_(A*N*HMA) values in going from the non-polar to the polar–aprotic solvents, corresponding to bathochromic shifts in the maximum of the observed bands (associated with a reduction of the *E*_T_(A*N*HMA) values), resulting from a higher polarizability and H-bond acceptor ability of the solvent, while those observed in going from the polar–aprotic to the polar–protic solvents (corresponding to hypsochromic shifts, associated with an increase of the *E*_T_(A*N*HMA) values) are dominated by the H-bond donor ability of the solvent. Overall, these changes in the dominant solvent effects in non-polar and polar–aprotic solvents on one side and in a polar–protic solvent, on the other side, explain the reversal in solvatochromism observed for the studied compound. The dipolarity term has a residual relevance in the case of band II, being relatively more important in the case of band I. The dipolarity term is more relevant for the polar solvents (see Table 2) and since, in this case, the coefficient *e* is negative, this term contributes the most to reducing the *E*_T_(A*N*HMA)^I^ values of polar (both aprotic and protic) solvents and, thus, to distinguish the effect of these solvents on the position of the maximum of band I from that of the non-polar solvents.

It is also interesting to mention that wavelengths corresponding to *E*_T_(A*N*HMA)_0_^II^ and *E*_T_(A*N*HMA)_0_^I^ values in Equations (3) and (4) are 297.2 (±10.2) and 281.7 (±2.1) nm, respectively. These values are hypsochromically shifted in comparison with the observed position of the bands in all solvents and are then in consonance with the predicted relevance of the effect due to the polarizability of the solvent in all types of solvents. Indeed, the coefficient *d* affecting the term of polarizability of the solvent in the Catalán equation, has the largest absolute value among all coefficients in the model developed based on the band II data (Equation (3)), being also important, according to Equation (4), as a contributor to shift hypsochromically band I even for the most non-polar solvents, cyclohexane and *n*-hexane, for which the Catalán dipolarity and H-bond donor parameters, SdP and SA, are zero, and the H-bond acceptor parameter SB is also very small.

**Figure 3:**
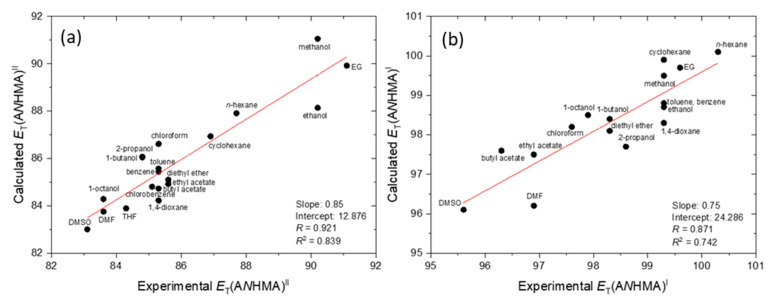
Plots of calculated *E*_T_(A*N*HMA)^II^ (**a**) and *E*_T_(A*N*HMA)^I^ (**b**) using the developed Catalán models as a function of the corresponding *E*_T_(A*N*HMA) values.

Figure 3 shows plots of the *E*_T_(A*N*HMA) values calculated from the developed Catalán models vs. experimental values, which highlight the fairly good general predictive ability of the models but also show that specific solute–solvent interactions are also relevant for several of the solvents, which limit in some extent the goodness of the fits, as revealed by the *R*^2^ values of the fittings, in particular for band I whose maximum of absorption varies the least.

### 2.3. DFT and TD-DFT Calculations: Structures and Spectra of Isomeric Species of ANHMA

**Scheme 1:**
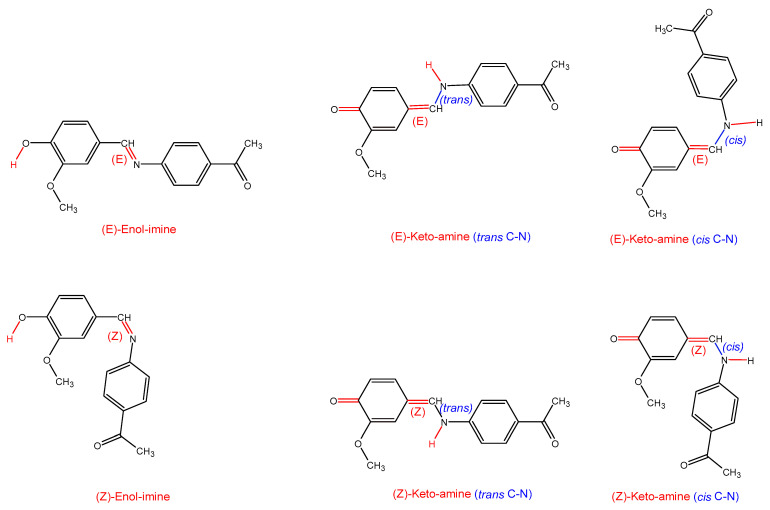
*E*- and *Z*- forms of enol-imine and keto-amine tautomers of A*N*HMA.

A*N*HMA has four major basic isomeric forms, which are depicted schematically in Scheme 1: two enol-imine forms with (*E*) and (*Z*) configuration about the imine C=N bond, and two keto-amine forms with (*E*) and (*Z*) arrangements around the exocyclic C=C bond. All these species have several conformers that result from the different possible orientations of the OH, OCH_3_, C(=O)CH_3_ substituents and of the whole hydroxy-methoxyphenyl and acetylphenyl fragments, in the case of the enol-imine forms, and from the different orientations of the OCH_3_ and C(=O)CH_3_ substituents and of the whole acetylphenyl and acetylanilino fragments, in the case of the keto-amine forms. The DFT(B3LYP)/6-311++G(d,p) structures of the conformers of the different forms are provided in the Supporting Information Figures S1–S4 and their relative energies (in the gas phase) are given in Table S1.

The calculations indicate that in the gas phase, the (*E*)-enol-imine form is the most stable species, with the (*Z*)-enol-imine conformers being at least 22 kJ mol^−1^ higher in energy, and all keto-amine species having energies at least 42 kJ mol^−1^ above that of the most stable conformer of the (*E*)-enol-imine form. These results are in consonance with the (*E*)-enol-imine form being the species existing in the synthesized polycrystalline material, as indicated by its IR spectrum (Figure S5).

The (*E*)-enol-imine form of A*N*HMA is predicted by the calculations to possess 16 different conformers. The four lowest energy conformers can be grouped in two pairs of nearly degenerated conformers ((*E*)-EI1, (*E*)-EI2, and (*E*)-EI3, (*E*)-EI4), with the conformers of the second pair having energies ca. 5–6 kJ mol^−1^ above those belonging to the first pair (see Table S1). These four low-energy conformers are stabilized by an intramolecular OH···OCH_3_ hydrogen bond, the calculated H···O hydrogen bond distance in these conformers being in the range 2.088–2.099 Å. The lowest energy pair of conformers ((*E*)-EI1, (*E*)-EI2) have the methoxy substituent and the nitrogen atom of the imine bridge pointing to the same side, while the second pair of conformers ((*E*)-EI3, (*E*)-EI4) have these groups pointing to opposite sides; the conformers in each pair differ in the relative orientation of the acetyl substituent (see Figure S1). The relative energies of the remaining (*E*)-enol-imine conformers, which differ from the most stable ones by the orientation of the OH and OCH_3_ groups, have calculated energies in the range of ~20–30 kJ mol^−1^ compared to that of the most stable conformer.

In the (*E*)-enol-imine conformers, the two rings make an angle of 43–45°, all forms having a symmetry-equivalent structure where the rings are rotated to the opposite direction around the N–C aniline bond. The acetyl and hydroxyl substituents are always aligned with the corresponding rings, while the methoxy substituent stays aligned with the phenolic ring in the first eight conformers in order of energy and is tilted ca. 67–70° from the plane of the ring in the last eight conformers.

The conformers of (*Z*)-enol-imine form have structures where the two rings are nearly perpendicular (to within 5°) to minimize steric repulsions (which are the major reason for the considerably higher energy of these forms compared to the (*E*)-enol-imine structures). Two (*Z*)-enol-imine conformers have an intramolecular OH···OCH_3_ hydrogen bond and have energies ca. 22 and 27 kJ mol^−1^ above that of the most stable conformer of the (*E*)-enol-imine form ((*E*)-EI1), all the other conformers having much higher energies (above 42 kJ mol^−1^, compared (*E*)-EI1). In the four lower energy (*Z*)-enol-imine conformers, the substituents attached to the rings are aligned with the corresponding ring plane, while the remaining four conformers have the methoxy group deviated ~67–69° out of ring plane, all conformers being doubly-degenerated by symmetry (Figure S2).

The conformers of the (*Z*)-keto-amine tautomer can be divided into two groups, depending on the arrangement about the central C(H)–N bridging bond. The C-N *trans* conformers have substantially lower energy than the C-N *cis* ones (~42–49 kJ mol^−1^, vs. ~70–77 kJ mol^−1^ relatively to the most stable (*E*)-enol-imine form) due to the strong steric repulsions in the latter (see Figure S3). The common characteristic of these two groups of conformers is that within each group, the energies of the conformers are quite similar: in the C-N *trans* group of conformers, the energies vary within 4 kJ mol^−1^, with the two most stable members with energies differing less than 1 kJ mol^−1^; in the C-N *cis* group, the numbers are similar (see Table S1). Two of the four (*Z*)-keto-amine C-N *trans* conformers have a planar skeleton and correspond to single structures (C_s_ symmetry), while in the remaining two conformers, the methoxy substituent is tilted out of the ring plane by ca. 43° and have one symmetry-equivalent form each. In turn, all six (*Z*)-keto-amine C-N *cis* conformers are non-planar due to the steric repulsions between the two rings (see Figure S6) and correspond to pairs of symmetry-equivalent structures. In these forms, the C_ph_=C–N–C_ph_ central bridge dihedral angle is within the 27–35° range, and the C–N–C_ph_C_ph_ dihedral stays in the 20–27° range, while in the four of these conformers where the methoxy group is out of the ring plane, the C_ph_C_ph_–O–C(H_3_) dihedral angle is within the 43–46° range. 

The conformational space of the (*E*)-keto-amine tautomer is similar to that of the (*Z*)-keto-amine one, with C-N t*rans* and C-N *cis* conformers having energies ~48–54 kJ mol^−1^ and ~76–80 kJ mol^−1^ relatively to the most stable (*E*)-enol-imine form. Also, like for (*Z*)-keto-amine, the conformers of each of the two groups of conformers of the (*E*)-keto-amine tautomer have very similar energies (equal to within ca. 4 kJ mol^−1^, with the two most stable members of each group differing in energy by less than ca. 1 kJ mol^−1^). The geometries of the C-N *trans* and C-N *cis* conformers of the (*E*)-keto-amine tautomer (Figure S4) also follow the same trends as the equivalent conformers of (*Z*)-keto-amine. The molecular skeleton of the four C-N *trans* conformers is very close to planarity (both the C_ph_=C–N–C_ph_ central bridge and C–N–C_ph_C_ph_ dihedral angles are tilted by less than ~3° from planarity), with the methoxy group in two of these conformers being ~53° tilted out of the ring plane. These latter conformers are doubly-degenerated by symmetry, while the remaining two correspond to single structures. The six (*E*)-keto-amine C-N *cis* conformers are non-planar doubly-degenerated structures, where the C_ph_=C–N–C_ph_ central bridge dihedral angle stays within the 28–34° range, the C–N–C_ph_C_ph_ dihedral angle in the 22–29° range, and the C_ph_C_ph_–O–C(H_3_) dihedral angle is within the 52–54° interval in the four conformers where the methoxy group is out of the ring plane.

The dipole moments of the different forms were also calculated (gas-phase values; Table S1). The range of values of the dipole moments for (*E*)-enol-imine, (*Z*)-enol-imine, and both (*Z*) and (*E*)-keto-amine C-N *trans* conformers are within 4.0 and 6.0 Debye, with very few exceptions, while those calculated for the high-energy keto-amine C-N *cis* conformers are larger (5–10 Debye).

Calculations for the most stable forms of the (*E*)-enol-imine and (*Z*)-keto-amine tautomers in DMSO, chloroform, and methanol solution using the integral equation formalism variant of the polarized continuum model (IEFPCM), within the self-consistent reaction field (SCRF) theoretical framework [47,48], were undertaken to evaluate the influence of the non-specific effects of these solvents on the relative energies of these forms. Compared to the gas phase, the relative energy of the keto-amine forms reduces to about a half (from ~40 kJ mol^−1^ to ca. 20 kJ mol^−1^) in the case of methanol and DMSO and to ca. 30 kJ mol^−1^ in chloroform. We also tried to estimate the effect of the specific interactions (H-bonds or H-bond-like interactions) between the molecules of these solvents and the two tautomeric forms of the studied compound. The procedure used allows only a very rough estimation of these effects and is described in Figure S6. It essentially used average values of the different possible types of H-bond (or H-bond-like) interactions and calculated for each molecule the change in energy resulting from establishing intermolecular interactions at the expense of intramolecular ones. Then, the difference between these values for the different solvents is calculated. This very approximate procedure revealed that the keto-amine tautomer is stabilized relatively to the (*E*)-enol-imine form in all the solvents examined so that the energies of the two tautomers become very similar. This allows us to predict that, if a solvent-assisted mechanism exists that allows for proton transfer from the OH phenol moiety of the enol tautomer to the imine group (and vice versa), the keto-imine forms may be formed and exist in some amount in solution. For that, protic solvents and non-protic polar solvents that can act as proton acceptors immediately appear as suitable candidates to favor the possibility of experimental observation of the keto-amine forms. As shown in Section 2.4, this is indeed the case.

It shall also be mentioned that other tautomeric species might be conceivable for the basic A*N*HMA structure, for example those associated with the keto-enol tautomerism within the acetyl substituent (CH_3_C=O vs. CH_2_=COH) or with the (keto-imine)-(enol-amine) tautomerism involving the acetyl substituent and the imine bridge, but these forms are not expected to be important in the present context, since enolization of the acetyl moiety requires strong acid catalysis to be formed [49]. B3LYP/6-311++G(d,p) calculations performed on the enols of the acetyl tautomeric form derived from the most stable conformers of A*N*HMA (*E*)-enol-imine ((*E*)-EI1) and (*Z*)-keto-amine C-N *trans* forms revealed that the energies of these two species are over 150 and 60 kJ mol^−1^ above that of (*E*)-EI1.

**Figure 4:**
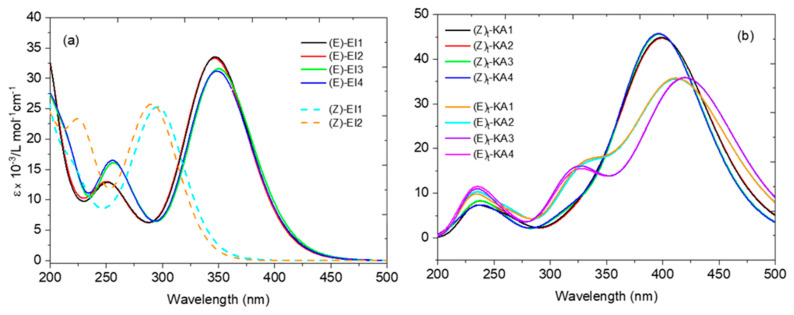
TD-DFT(B3LYP)/6-311++G(d,p) calculated UV-vis absorbance spectra of relevant A*N*HMA isomeric forms (gas phase): (*E*)- and (*Z*)-enol–imine lowest energy conformers (**a**), and (*Z*)- and (*E*)-keto–amine C–N *trans* conformers (**b**).

Figure 4 shows the TD-DFT(B3LYP)/6-311++G(d,p) calculated UV-vis spectra for selected low-energy conformers of the different A*N*HMA isomeric species.

According to the calculations (Tables S2–S5), in the gas phase, the longest wavelength absorption maxima in (*E*)-enol-imine and (*Z*)-enol-imine forms showing significant oscillator strength are predicted at 343–354 nm (HOMO → LUMO) and 292–303 nm (HOMO → LUMO+1 or HOMO–1 → LUMO, depending on the specific conformer), respectively, while those of the (*Z*)-keto-amine and (*E*)-keto-amine forms are predicted at 397–407 nm (HOMO → LUMO) and 410–434 nm (HOMO → LUMO), respectively. Considering only the C-N *trans* conformers of the keto-amine tautomeric forms, the wavelength ranges reduce to 397–399 nm ((*Z*)-keto-amine forms) and 410–420 nm ((*E*)-keto-amine forms). As shown in Figure 4, shorter wavelength bands are predicted for the different species above 200 nm. These bands include contributions from several transitions (in Tables S2–S5, only those involving the S_2_ and S_3_ states are indicated, but states up to S_20_ have predicted energies that stay in this range). For the (*E*)-enol-imine form, one band resulting from all these possible contributions is observed above 250 nm, which, according to the calculations, stays nearly at the same position as one of the two bands originated in other than the transition to S_1_ predicted for the keto-amine tautomers (both (*Z*) and (*E*) forms). TD-DFT calculations performed using solvent simulation (IEFPCM method) for the specific cases of chloroform, methanol, and DMSO indicate, in agreement with the solvatochromic results presented in Section 2.1 and Section 2.2, that the lowest energy transition of the (*E*)-enol-imine form undergoes a batochromic shift in going from the gas phase to solution, from ca. 345 nm to ca. 365 nm (TD-DFT calculated values; the values predicted from the solvatochromic model based on the experimental data are from about 300 nm to ca. 330 nm) with the largest shift predicted (also in agreement with the observations) for DMSO (ca. 20 nm as calculated by the TD-DFT method; predicted by the solvatochromic model: ca. 40 nm). It shall, however, be noticed that, while the TD-DFT calculations with solvent simulation follow the general trend regarding the relative values of the band maxima in gas phase vs. solution, the predictions are far from being quantitative, as it could be anticipated because the theoretical model ignores the specific interactions between the solute and the solvent molecules (e.g., H-bonds), which have been shown to be very important for the studied system by the performed analysis based on the application of the solvatochromic Catalán model described above.

As a whole, the results of the calculations (relative energies, electronic spectra) provide strong evidence for the assignment of the two major bands observed experimentally, in the 285–300 nm (band I) and 310–345 nm (band II) ranges, to the (*E*)-enol-imine tautomer, with band II corresponding to the HOMO → LUMO (S_1_ ← S_0_) transition. The UV-vis spectral data thus indicate that the (*E*)-enol-imine tautomer strongly predominates (or is the unique form) in the different studied solvents.

### 2.4. NMR Investigation of the ANHMA Tautomerism in Solution

DMSO, methanol, and chloroform were chosen for a detailed investigation of the tautomerism of A*N*HMA in solution as representative solvents. DMSO was chosen because of its unique characteristics within the context of the present study (it is the solvent for which the observed reversal in solvatochromism takes place; see Section 2.1 and Section 2.2), and methanol as a typical polar–protic solvent. The reason for choosing also chloroform will become clear later on, but it was essentially motivated by the fact that this solvent was the one where the studied compound was found to be photochemically less stable (Section 2.5).

#### 2.4.1. A*N*HMA in DMSO

**Figure 5:**
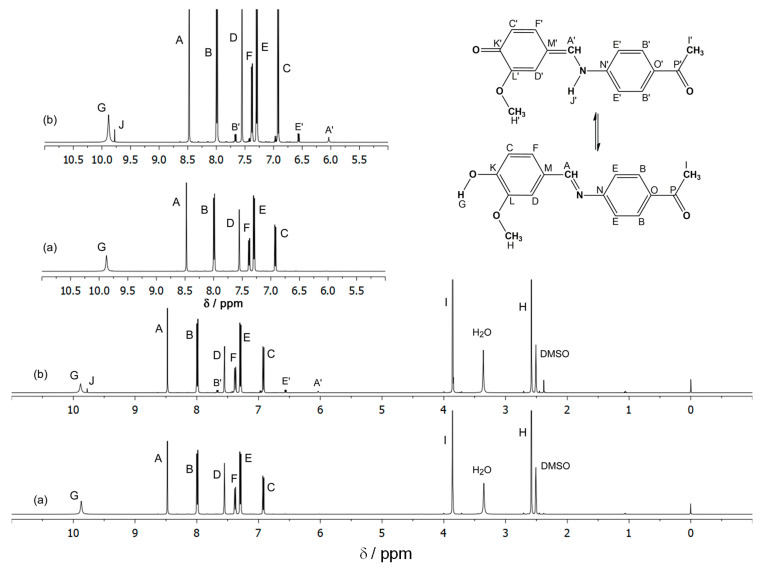
^1^H NMR spectra of A*N*HMA in DMSO-d_6_ obtained (**a**) immediately and (**b**) one week after the dissolution; one inset containing the expansion from 5.00 to 11.00 pm for better visualization of the proton signals for the predominant form of A*N*HMA (the enol-imine tautomer) (92%) and the minor signals assigned to an additional species (the keto-amine tautomer) (8%), in a slow exchange equilibrium, in the NMR time scale. The signals at 3.36 and 2.51 ppm are assigned to traces of H_2_O and DMSO, respectively.

**Figure 6:**
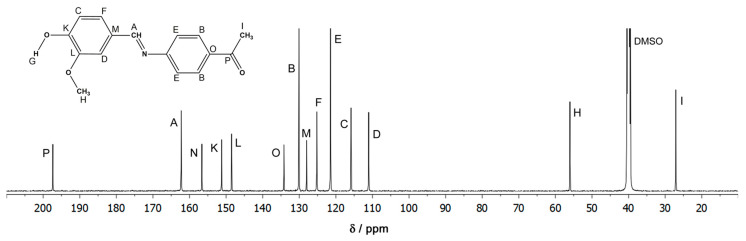
^13^C NMR spectrum of A*N*HMA in DMSO-d_6_. The signals detected are assigned to the predominant form (the enol-imine tautomer). The spectrum obtained one week after the preparation of the solution is not shown because the signals of the minor form (the keto-amine tautomer) are not detected in ^13^C NMR due to its low concentration. The signal detected at 40 ppm is assigned to DMSO.

Figure 5 and Figure 6 show the ^1^H and ^13^C NMR spectra of A*N*HMA in DMSO-d_6_ solution. The correspondent bidimensional spectra are shown in Figures S7–S10, and the ^1^H and ^13^C NMR chemical shifts are presented in Table 3 and Table 4, respectively, where they are compared with theoretically predicted values calculated at the GIAO/DFT(B3LYP)/6-311++G** level (where GIAO refers to the Gauge-Independent Atomic Orbital method [50,51]).

For a freshly prepared solution, the spectra exhibited only signals due to the (*E*)-enol-imine form, which confirmed the UV-vis data and was in agreement with the results of the calculations. We noticed, however, that with time, additional peaks emerged in the ^1^H spectrum. After one week of storage of the solution in the dark, the ^1^H spectrum shows a set of new peaks that can be ascribed as the keto-amine tautomer (8% vs. 92% of the enol tautomer). The OH proton of the (*E*)-enol-imine form is observed at 9.87 ppm, in the expected position for a phenolic OH proton in DMSO [52] (the calculated chemical shift in the gas phase is lower), as a relatively broad feature, while the emerging NH proton is observed at 9.78 ppm. The assignment of these peaks to the OH and NH protons is doubtless, taking into account also the results of the bidimensional spectra shown in the Supporting Information, while the assignment of the other peaks is straightforward. The observation of the keto-amino form in the DMSO solution after one week of storage demonstrates that the equilibrium between the two forms is very slow, which also indicates that the lability of the OH and NH protons is, in this solvent, very reduced. This also justifies the clear observation of the peaks due to these protons, which would not be possible if they were exchanging fast with solvent deuterium atoms. In fact, it seems very much possible that the formation of the keto-amine tautomer in DMSO is essentially due to the presence of traces of water (that could not be eliminated in spite of our efforts), suggesting that in the absence of it the (*E*)-enol tautomer would be the sole form present in solution.

#### 2.4.2. A*N*HMA in Methanol

**Figure 7:**
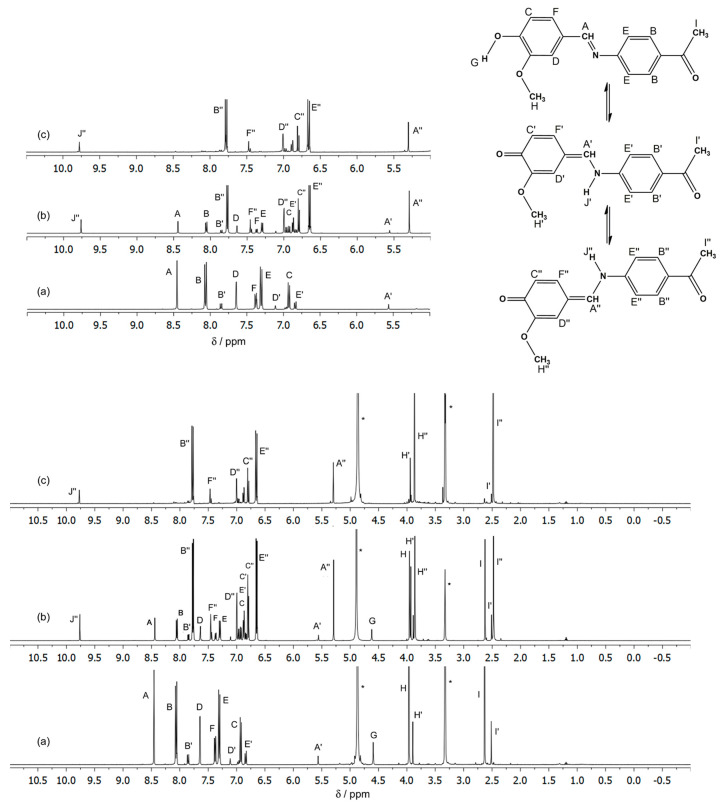
^1^H NMR spectra of A*N*HMA in CD_3_OD obtained (**a**) immediately, (**b**) 1 h after the preparation, and (**c**) after the *λ* ≥ 311 nm irradiation of the initial solution (>30 min); one inset containing one expansion from 5.00 to 11.00 pm for better visualization of the signals. The signals at 3.35 and 4.87 ppm (*) are assigned to the residual protons of methanol.

**Figure 8:**
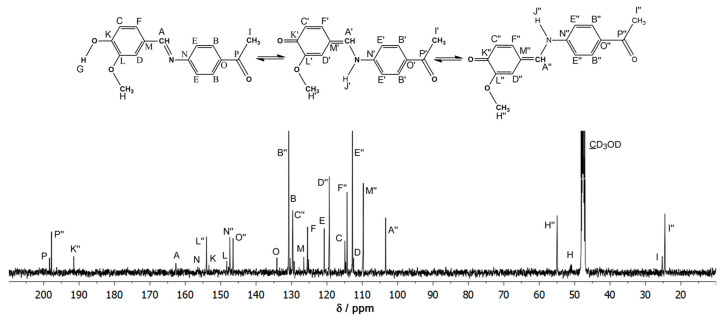
^13^C NMR spectrum of A*N*HMA in CD_3_OD obtained 1 h after the preparation. The signals have been assigned to a mixture of the keto-amine tautomeric forms and the most abundant enol-imine tautomer. The signal detected at 48 ppm is assigned to the solvent.

Figure 7 and Figure 8 show the ^1^H and ^13^C NMR spectra of A*N*HMA in a methanol-d_4_ solution. The correspondent bidimensional spectra are shown in Figures S11–S14. The ^1^H and ^13^C NMR chemical shifts are given in Table 3 and Table 4.

The ^1^H NMR spectrum obtained immediately after the dissolution (lower spectrum in Figure 7) shows as the predominant species the (*E*)-enol-imine tautomer, but low-intensity peaks due to the (*Z*)-keto-amine form are also visible, demonstrating that this species is also present in the studied solution. After 1 h of storage of the solution in the dark, the ^1^H NMR spectrum changed considerably, with two sets of bands ascribable to the (*Z*)- and (*E*)-keto-amine forms being clearly visible, while those originated in the (*E*)-enol-imine tautomer reduced of intensity. In this (protic) solvent, the enol-imine/keto-amine equilibrium is attained more easily than in DMSO since the solvent can mediate the proton transfer. Nevertheless, the fact that both OH and NH protons can be observed in their expected positions (note that in this case, the OH proton appears at a much lower chemical shift—at 4.60 ppm—than in DMSO, as usually occurs for phenols [48]), reveals that the two protons are still not exchanging very much efficiently. In the case of the OH proton, this may indicate that the intramolecular hydrogen bond in the (*E*)-enol-imine tautomer subsists in significant amounts in methanol solution.

Another interesting observation is that both (*Z*)- and (*E*)-keto-amine forms participate in the equilibrium. There is nothing that can be expected to preclude the formation of both keto-amine isomers from the (*E*)-enol-imine species (the two keto forms are just the geometrically most favored products for different starting conformations of the (*E*)-enol-imine form; on the other hand, they cannot interconvert because this would require surpassing a high energy barrier associated with the rotation around the C=C double bond), but since the gas-phase energy of the (*E*)-keto-amine form is somewhat higher in gas phase than that of the (*Z*) form, the results suggest the (*E*) isomer is somehow more stabilized in methanol (probably because of a slightly larger average dipole moment, when the lowest energy C-N *trans* conformers of the two keto-amine isomers are compared; see Table S1).

#### 2.4.3. A*N*HMA in Chloroform

**Figure 9:**
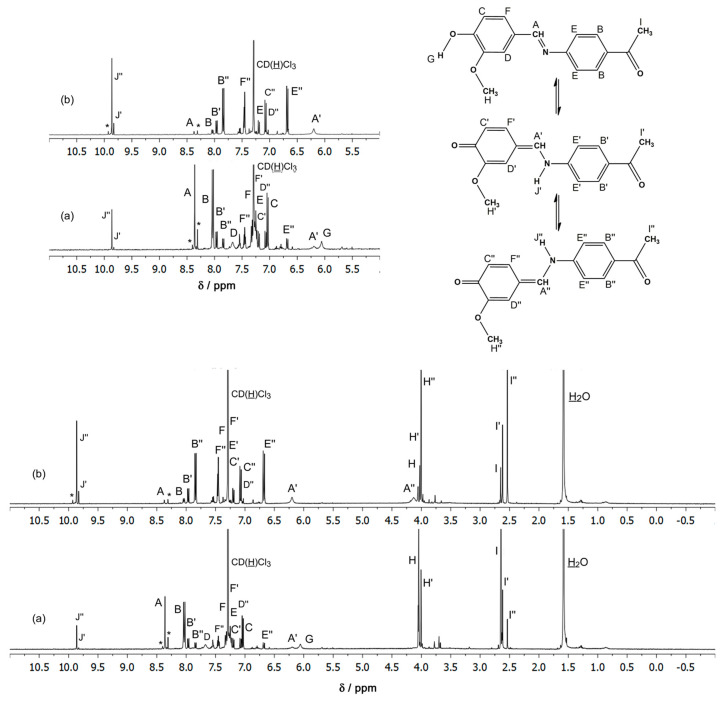
^1^H NMR spectra of A*N*HMA in CDCl_3_ obtained (**a**) immediately and (**b**) 1 h after the preparation; one inset containing the expansion from 5.00 to 11.00 ppm for better visualization of the signals for the forms of the compound present in solution, the (*E*)-enol-imine tautomer and both (*Z*)- and (*E*)-isomer of the keto-amine tautomer, in a slow exchange equilibrium in the NMR time scale. Minor signals marked with * have uncertain origins. The signals at 1.58 and 7.29 ppm are assigned to traces of H_2_O and residual protons of CHCl_3_.

Figure 9 shows the ^1^H spectra of A*N*HMA in the CDCl_3_ solution. The bidimensional spectra are shown in Figures S15–S18, and the chemical shifts are presented in Table 3.

Like in methanol solution, in a freshly prepared chloroform solution, the compound exists predominantly in the (*E*)-enol-imine tautomeric form, with the keto-tautomer (in both (*Z*) and (*E*) isomeric forms) also present in a significant amount. In addition, like for the other solutions investigated, the amount of keto-amine tautomer increases with the time of storage of the solution (in the dark) so that after ca. 1 h, this tautomer appears as the major constituent. This result is interesting to note because this can be taken as an indication that in this solution (as well as in methanol, where also after 1 h the keto-amine tautomer seems to be dominant), the keto-amine tautomer might be slightly more stable than the (*E*)-enol-imine form. The equilibrium is reached slowly in all cases, starting from the (*E*)-enol-imine form that is present in the solid before dissolution. In the DMSO solution, the enol form seems to be the most stable one. These results are also in agreement with the predicted most significant stabilization in methanol and chloroform of the keto-imine tautomer than in DMSO, in particular, due to specific interactions between the molecules of the first two solvents and the dye (see Figure S6).

### 2.5. Photoisomerization (λ ≥ 311 nm) of ANHMA in Different Solvents

UV (*λ* ≥ 311 nm) irradiation of A*N*HMA (for a maximum of 30 min) in all studied solvents was undertaken. Significant changes in the compound’s UV-vis absorbance spectrum were only observed for solutions of the compound in chlorobenzene and chloroform (both chlorinated polar–aprotic solvents) and to a lesser extent in *n*-butyl acetate (polar–aprotic), and 2-propanol, 1-butanol, ethanol, and methanol (polar–protic). The results obtained in chlorobenzene and chloroform solutions were distinct because these were the only cases where the solution considerably changed its visual appearance. Being initially colorless and transparent, the solution quickly turned to light yellow (in the first case) or became translucent (chloroform). All other solutions are colorless and transparent after exposure to irradiation.

**Figure 10:**
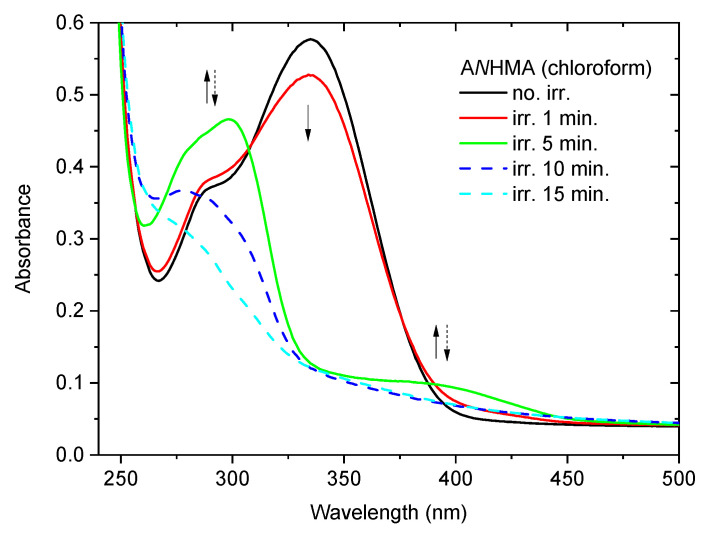
Absorbance spectrum of A*N*HMA in chloroform solution as a function of time of UV irradiation (*λ* ≥ 311 nm).

The changes in the UV-vis spectrum of A*N*HMA in chloroform solution along irradiation are shown in Figure 10. After 1 min of irradiation, a reduction of the intensity of the band II (*λ*_max_^II^ = 335 nm), ascribed to the HOMO → LUMO transition of the (*E*)-enol-imine, was observed. The change of the intensity of band I (*λ*_max_^I^ = 293 nm; also due to the (*E*)-enol-imine form) is difficult to judge because new bands emerge at nearby the same position. Globally, the intensity within the wavelength range 270–320 nm increases, obscuring the expected decrease of intensity of band I. Concomitantly, a broad band appears in the 370–440 nm region (with an absolute maximum at ca. 390 nm; band III). The bands undergoing intensity fit well the predictions for the bands of the keto-amine isomeric forms (see Figure 4), and the broadness of band III can be easily correlated with the simultaneous contribution to this band of the (*Z*)- and (*E*)-isomeric forms of the keto-amine tautomer. Hence, it can be concluded that irradiation starts by promoting the photochemical conversion of the (*E*)-enol-imine tautomer into the keto-amine forms. After 5 min of irradiation, these changes were observed to attain their maximum, and from that time on, all bands in the spectra ascribable to A*N*HMA reduced intensity, indicating photodegradation of the compound upon prolonged UV irradiation.

The UV irradiation experiments carried out in chloroform solution were also attempted to be followed by NMR. However, the fast degradation of the compound upon irradiation and also the most difficult characterization by this technique of the compound in this solvent (including the fact that solvent bands appear in spectroscopically relevant spectral regions) posed practical difficulties that could not be overcome, precluding this investigation.

**Figure 11:**
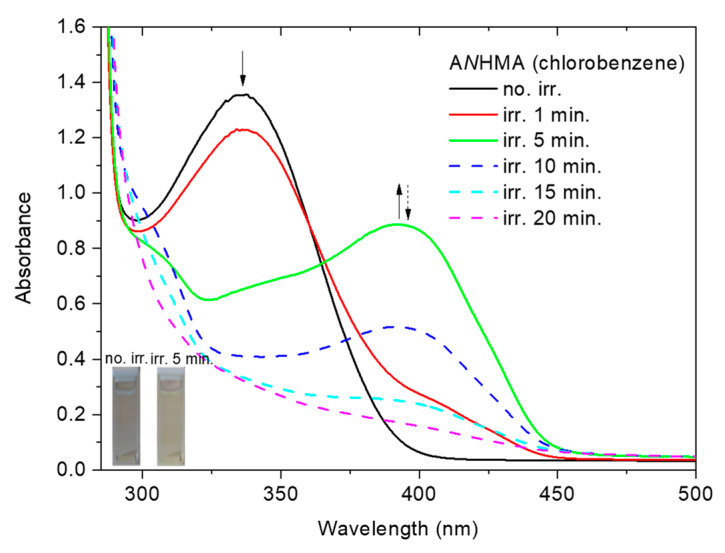
Absorbance spectrum of A*N*HMA in chlorobenzene solution as a function of time of UV irradiation (*λ* ≥ 311 nm).

The results obtained in chlorobenzene are shown in Figure 11. In this solvent, band I, due to (*E*)-enol-imine tautomer, is not seen due to the overlap with a solvent band, but the changes in band II are clear: upon irradiation, this band reduces in intensity, while band III, ascribed to the keto-amine forms grows during the first 5 min of irradiation, indicating occurrence of phototransformation of the (*E*)-enol-imine tautomer into the keto-amine forms, as also observed in chloroform solution. This reaction, and also the (*Z*) to (*E*) C=C photoisomerization between the keto-amine isomers, takes place rather extensively and justifies the observed change of the color of the solution to yellow (see insert in Figure 11). Prolonged irradiation was found to lead to photodegradation of the compound, with all bands due to A*N*HMA reducing in intensity.

**Figure 12:**
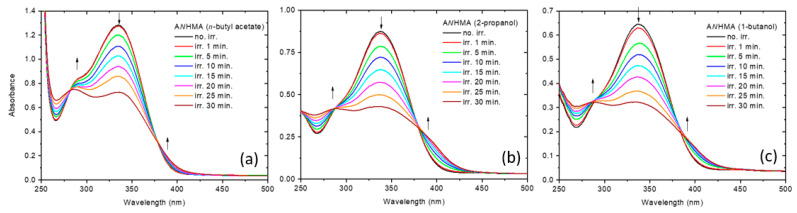
Absorbance spectrum of A*N*HMA in *n*-butyl acetate (**a**), 2-propanol (**b**), and 1-butanol (**c**) solutions as a function of time of UV irradiation (*λ* ≥ 311 nm).

The photolysis results obtained in *n*-butyl acetate, 2-propanol, and 1-butanol are similar and follow the general trend observed for the two chlorinated solvents described above, i.e., irradiation promotes the conversion of (*E*)-enol-imine tautomer into both structural isomers of the keto-amine tautomer (Figure 12), but the efficiency of the processes, is lower.

**Figure 13:**
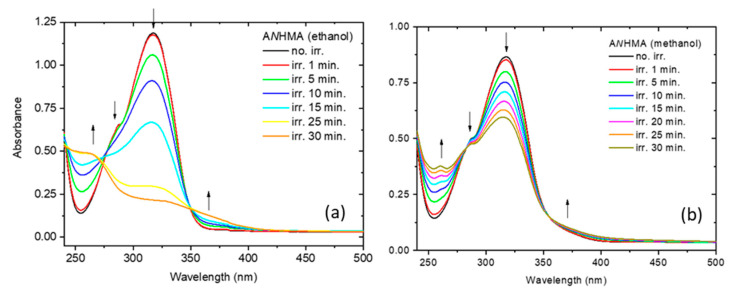
Absorbance spectrum of A*N*HMA in ethanol (**a**) and methanol (**b**) as a function of time of UV irradiation (*λ* ≥ 311 nm).

In the most polar and acidic solvents, ethanol and methanol, the investigation of the photochemistry of A*N*HMA revealed essentially the same panorama as for the other studied solvents. In this case, upon UV irradiation, the (*E*)-enol-imine bands (both bands I and II) started to decrease visibly in intensity from the very beginning of irradiation (Figure 13), while the bands ascribed to keto-amine forms in the shorter wavelength range and band III increase of intensity. The efficiency of the processes is similar to those observed for the other alcohols. The experiment in methanol was followed by ^1^H NMR. For that, photolysis was performed on the compound in a UV-vis transparent tube. The data confirm the general conclusions extracted from the UV-vis spectra but demonstrate that in this case, after prolonged irradiation (>30 min), the (*E*)-keto-amine isomer is the strongly dominant product (see Figure 7c). In this regard, the photochemically-induced process resembles the thermal process followed by NMR spectroscopy, and described above, where the solution was monitored along the storage time in the dark, which also tends to result in the (*E*)-keto-amine form as the most abundant species in methanol. However, the photochemical processes are much faster and more efficient.

## 3. Materials and Methods

### 3.1. Synthesis and Characterization (IR and NMR)

**Scheme 2:**
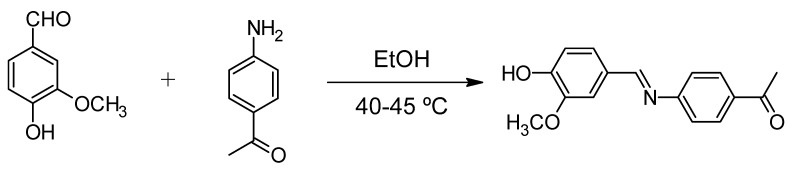
Synthesis route for A*N*HMA.

(*E*)-4-acetyl-*N*-(4-hydroxy-3-methoxybenzylidene)aniline (A*N*HMA) was synthesized from 4-hydroxy-3-methoxybenzaldehyde and 4-acetylaniline (Scheme 2), following a general procedure previously reported [28,53,54,55,56,57]. 4-Hydroxy-3-methoxybenzaldehyde (1.521 g and 0.01 mol) and 4-acetylaniline (1.352 g and 0.01 mol) were first dissolved in 25 mL ethanol in separate beakers at 40–45 °C, and the solutions were then mixed slowly. After a precipitate was formed, the mixture was stirred for another 1 h at the same temperature. The precipitated product was left cooling for 2–3 h at room temperature, and then it was filtered, purified by recrystallization from ethanol, and dried in a vacuum desiccator at room temperature. Its purity was checked by thin layer chromatography (TLC) in ethyl acetate: *n*-hexane (2:1). The infrared (IR) spectrum of the purified polycrystalline material (in a KBr pellet) and the ^1^H- and ^13^C-NMR spectra in deuterated dimethylsulfoxide (DMSO-d_6_) solution were found to be compatible with the desired product (Supporting Information Figures S5, S7 and S8), the IR data demonstrating that in the solid phase the compound exists in its enol-imine tautomeric form:

A*N*HMA. IR (KBr pellet, ν/cm^−1^): 3315 (O-H, H-bonded), 3083, 3029 (C-H, aromatic), 2972, 2940, 2904, 2875 (C-H, aliphatic), 1668 (C=O), 1583 (C=N), 1512–1463 (C=C, aromatic), 1390 (COH bend), 1274/1028 (C-O-C, alkyl aryl ether), 1250 (C-O phenol), 683 (OH torsion, phenol). ^1^H NMR (400 MHz, DMSO-d_6_, δ/ppm; (*E*)-enol-imine tautomer): 9.87 (s, 1H), 8.48 (s, 1H), 7.99 (d, *J* = 8.3 Hz, 2H), 7.55 (s, 1H), 7.38 (d, *J* = 8.2 Hz, 1H), 7.30 (d, *J* = 8.3 Hz, 2H), 6.92 (d, *J* = 8.1 Hz, 1H), 3.85 (s, 3H), 2.58 (s, 3H). ^13^C NMR (101 MHz, DMSO-d_6_, δ/ppm): 197.36 (s), 162.26 (s), 156.62 (s), 151.22 (s), 148.51 (s), 134.39 (s), 130.09 (s), 128.00 (s), 125.20 (s), 121.47 (s), 115.85 (s), 111.02 (s), 56.03 (s), 27.09 (s).

The IR spectrum was recorded in a Perkin–Elmer FTIR 100 spectrometer. The ^1^H and ^13^C NMR spectra were collected in deuterated DMSO, CD_3_OD, and/or CDCl_3_ solutions using a Bruker Avance III HD 500 MHz NMR spectrometer. The ^13^C spectra were recorded using proton decoupling techniques, taking advantage of the nuclear Overhauser effect. The ^1^H and ^13^C NMR signals of the solvents were used as internal references for ^1^H (δ 2.51, 3.35, 4.87, and 7.27 ppm) and ^13^C (δ 40, 48, and 77.23 ppm) chemical shifts, respectively. The homo- and heteronuclear 2D NMR spectra, COSY, NOESY, HSQC, and HMBC were recorded on the same spectrometer. The assignments of the ^1^H and ^13^C NMR signals detected are based on bidimensional experiments, COSY, NOESY, HSQC, and HMBC, and compared with the calculated values. All experiments were performed at room temperature

### 3.2. UV-Vis Absorption Spectra

All solvents used in the experiments are of high purity and spectroscopic grade and were purchased from Sigma-Aldrich. The concentrations of the A*N*HMA solutions were kept as low as possible (<3 ×10^−4^ M) in order to avoid aggregation of the compound. The ultraviolet-visible (UV-vis) spectra were obtained in the 200–700 nm wavelength range using a Perkin–Elmer Lambda-35 UV-vis spectrometer. All measurements were performed using a quartz cell (standard cell, with 1 cm × 1 cm optical path) at room temperature, and then the solutions were kept in the dark for 5 days after preparation. Whenever required, the maximum wavelength values of the absorption bands were determined after band deconvolution, performed using OriginPro 2021 [58].

### 3.3. Quantum Chemical Calculations

All calculations were carried out at the DFT/B3LYP level of theory, with the 6-311++G(d,p) basis set [59,60,61,62,63,64] using GAUSSIAN 09 (revision C.01) [65]. Possible isomeric structures of A*N*HMA were subjected to geometry optimization in their electronic ground state, and their relative energies and vibrational frequencies (calculated using the same method and basis set) were obtained. The lack of imaginary frequencies indicates that all optimized geometries correspond to true minima. Excited state calculations were undertaken within the time-dependent DFT (TD-DFT) theoretical framework [66,67] for the isolated molecule *in vacuo* using the same functional and basis set as for the ground state calculations. The bulk solvent effects of chloroform, methanol, and DMSO were considered within the polarizable continuum model (PCM) framework using the integral equation formalism variant (IEFPCM) [47,48]. The NMR predicted data were obtained using the Gauge-Independent Atomic Orbital (GIAO) approach [50,51] at the B3LYP/6-311++G(d,p) level of theory in the different solvents. The ^1^H and ^13^C calculated GIAO absolute shieldings (σ; ppm) were converted into chemical shifts relative to tetramethylsilane (TMS) (δ values; ppm) by subtraction from the tetramethylsilane (TMS) GIAO calculated absolute shieldings.

The GaussView 5.0 [68] and ChemCraft (version 1.8) [69] programs were used both for input structure generation and visualization of the results.

## 4. Conclusions

In this study, a novel benzylidene aniline Schiff base derivative was synthesized and characterized using infrared and ^1^H- and ^13^C-NMR spectroscopies. The solvatochromic behavior of the compound was studied in 19 solvents with different characteristics. UV-vis absorption data analysis was done using the Catalán solvatochromic methodology and was supported by the thorough theoretical investigation of the structural characteristics of the different tautomeric/isomeric forms of the compound.

As for a previous bis-azo dye based on naphthalen-1-amine recently studied in our laboratories [5], the compound was shown to exhibit a reversal in solvatochromism for solvent Reichardt’s *E*_T_(30)~45 kcal mol^−1^ (DMSO), as a consequence of the change in the dominant solvent effects in non-polar and polar–aprotic solvents (H-bond acceptor ability of the solvent and polarizability) compared to polar–protic solvents (H-bond donor ability), as revealed by the Catalán analysis.

In all freshly prepared solutions of A*N*HMA, the (*E*)-enol-imine tautomeric form strongly dominates (or occurs exclusively), as shown by the UV-vis and NMR spectroscopic data. Upon irradiation of the A*N*HMA solutions with UV light (*λ* ≥ 311 nm), the compound undergoes solvent-mediated (*E*)-enol-imino → keto-amino tautomerization, as well as (*Z*)-keto-amino → (*E*)-keto-amino C=C isomerization, the latter process being suggested by accumulation of the (*E*)-keto-amino form with time of irradiation. The photoreactions are particularly fast in chlorobenzene and chloroform, both chlorinated polar–aprotic solvents, and less effective in *n*-butyl acetate (polar–aprotic), and 2-propanol, 1-butanol, ethanol, and methanol (polar–protic), which may be considered an indication that triplet states might participate in the photoprocesses, since the ability of chlorine atoms to stabilize triplet states is well-known, which makes them energetically more accessible through spin-orbit coupling [70], but this conclusion requires confirmation (e.g., using time-resolved spectroscopy).

The studied molecule proved to be a versatile compound whose optical properties are very sensitive to solvent properties, which can be taken advantage of for its potential uses in different areas, such as sensing and detection, optoelectronic devices, and smart materials.

## Figures and Tables

**Table 1:** Reichardt’s solvent parameter (*E*_T_(30), kcal mol^−1^) for the used solvents, maximum UV absorption wavelengths (*λ*_max_, nm) of A*N*HMA in the different solutions, and *E*_T_ solvent parameter obtained from the spectroscopic data of A*N*HMA (*E*_T_(A*N*HMA), kcal mol^−1^).

N	Solvent	*E*_T_(30)	*λ*_max_^I^	*E*_T_(A*N*HMA)^I^	*λ*_max_^II^	*E*_T_(A*N*HMA)^II^
1	cyclohexane	30.9	288	99.3	329	86.9
2	*n*-hexane	31.0	285	100.3	326	87.7
3	toluene	33.9	288	99.3	335	85.3
4	benzene	34.3	288	99.3	335	85.3
5	diethyl ether	34.5	291	98.3	334	85.6
6	1,4-dioxane	36.0	288	99.3	335	85.3
7	chlorobenzene	36.8			336	85.1
8	THF	37.4			339	84.3
9	ethyl acetate	38.1	295	96.9	334	85.6
10	*n*-butyl acetate	38.5	297	96.3	335	85.3
11	chloroform	39.1	293	97.6	335	85.3
12	DMF	43.2	295	96.9	342	83.6
13	DMSO	45.1	299	95.6	344	83.1
14	1-octanol	48.1	292	97.9	342	83.6
15	2-propanol	48.4	290	98.6	337	84.8
16	1-butanol	49.7	291	98.3	337	84.8
17	ethanol	51.9	288	99.3	317	90.2
18	methanol	55.4	288	99.3	317	90.2
19	EG	56.3	287	99.6	314	91.1

*E*_T_(A*N*HMA) (kcal mol^−1^) = *hc*N_A_/(*λ*_max_, nm) = 28,591.5/(*λ*_max_, nm).

**Table 2:** Reichardt’s *E*_T_(30) (kcal mol^−1^) and Catalán solvent parameters for the used solvents [1,6].

		*E*_T_(30)	Catalán Parameters			
N	Solvent		SA	SB	SP	SdP
1	cyclohexane	30.9	0.000	0.073	0.683	0.000
2	*n*-hexane	31.0	0.000	0.056	0.616	0.000
3	toluene	33.9	0.000	0.128	0.782	0.284
4	benzene	34.3	0.000	0.124	0.793	0.270
5	diethyl ether	34.5	0.000	0.562	0.617	0.385
6	1,4-dioxane	36.0	0.000	0.444	0.737	0.312
7	chlorobenzene	36.8	0.000	0.182	0.833	0.537
8	THF	37.4	0.000	0.591	0.714	0.634
9	ethyl acetate	38.1	0.000	0.542	0.656	0.603
10	*n*-butyl acetate	38.5	0.000	0.525	0.674	0.535
11	chloroform	39.1	0.047	0.071	0.783	0.614
12	DMF	43.2	0.031	0.613	0.759	0.977
13	DMSO	45.1	0.072	0.647	0.830	1.000
14	1-octanol	48.1	0.299	0.923	0.713	0.454
15	2-propanol	48.4	0.283	0.830	0.633	0.808
16	1-butanol	49.7	0.341	0.809	0.674	0.655
17	ethanol	51.9	0.400	0.658	0.633	0.783
18	methanol	55.4	0.605	0.545	0.608	0.904
19	EG	56.3	0.717	0.534	0.777	0.910

**Table 3:** ^1^H NMR chemical shifts ^a^ for A*N*HMA in DMSO, CD_3_OD, and CDCl_3_ (298 K).

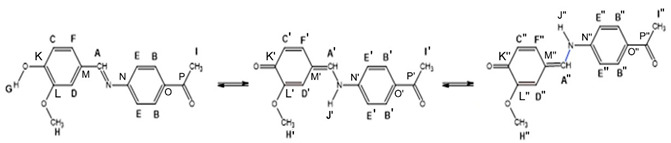										
DMSO										
	H(A)	H(B)	H(C)	H(D)	H(E)	H(F)	H(G)	H(H)	H(I)	
δ (*E*)-enol-imine	8.48 (8.56)	7.99 (8.48/8.31) [8.40]	6.92 (7.28)	7.55 (8.01)	7.30 (7.62/7.35) [7.48]	7.38 (7.50)	9.87 (6.27)	3.85 (4.14)	2.58 (2.69)	
	H(A’)	H(B’)	H(C’)	H(D’)	H(E’)	H(F’)		H(H’)	H(I’)	H(J’)
δ (*Z*)-keto-amine	6.02 (7.90)	7.66 (8.53/8.27) [8.40]	-(b) (6.58)	-(b) (7.57)	6.57 (7.70/7.33) [7.52]	-(b) (8.51)		3.86 (4.03)	2.38 (2.66)	9.78 (7.73)
CD_3_OD										
	H(A)	H(B)	H(C)	H(D)	H(E)	H(F)	H(G)	H(H)	H(I)	
δ (*E*)-enol-imine	8.44 (8.56)	8.05 (8.48/8.31) [8.40]	6.94 (7.28)	7.64 (8.01)	7.29 (7.62/7.35) [7.48]	7.37 (7.50)	4.60 (6.27)	3.98 (4.14)	2.63 (2.69)	
	H(A’)	H(B’)	H(C’)	H(D’)	H(E’)	H(F’)		H(H’)	H(I’)	H(J’)
δ (*Z*)-keto-amine	5.54 (7.73)	7.76 (8.54/8.26) [8.40]	6.93 (6.58)	7.45 (7.56)	6.85 (7.68/7.33) [7.50]	7.34 (7.49)		3.86 (4.04)	2.50 (2.65)	9.76 (7.70)
	H(A’’)	H(B’’)	H(C’’)	H(D’’)	H(E’’)	H(F’’)		H(H’’)	H(I’’)	H(J’’)
δ (*E*)-keto-amine	5.27 (7.85)	7.74 (8.53/8.20) [8.37]	6.78 (8.62)	6.98 (7.15)	6.85 (7.62/7.24) [7.43]	7.44 (7.80)		3.67 (4.91)	2.48 (2.62)	9.77 (7.65)
CDCl_3_										
	H(A)	H(B)	H(C)	H(D)	H(E)	H(F)	H(G)	H(H)	H(I)	
δ (*E*)-enol-imine	8.36 (8.48)	8.03 (8.50/8.19) [8.35]	7.03 (7.24)	7.67 (8.00)	7.19 (7.56/7.25) [7.41]	7.37 (7.41)	6.05 (6.13)	4.05 (4.10)	2.65 (2.63)	
	H(A’)	H(B’)	H(C’)	H(D’)	H(E’)	H(F’)		H(H’)	H(I’)	H(J’)
δ (*Z*)-keto-amine	6.20 (7.70)	7.95 (8.65/8.14) [8.35]	7.06 (6.54)	-(b) (7.32)	6.67 (7.54/7.16) [7.35]	7.30 (7.32)		4.12 (4.14)	2.54 (2.59)	9.83 (7.33)
	H(A’’)	H(B’’)	H(C’’)	H(D’’)	H(E’’)	H(F’’)		H(H’’)	H(I’’)	H(J’’)
δ (*E*)-keto-amine	4.13 (7.67)	7.83 (8.57/8.10) [8.33]	7.06 (6.61)	7.25 (7.04)	6.69 (7.52/7.09) [7.30]	7.45 (7.61)		4.03 (4.22)	2.54 (2.57)	9.86 (7.35)

^a^ δ values, in ppm, relative to Me4Si, using the signals of solvents, DMSO, CD_3_OD and CDCl_3_ (δ_H_ = 2.51, 3.35 and 7.29 ppm, respectively) as internal references. ^b^ not assigned.

**Table 4:** ^13^C NMR chemical shifts ^a^ for A*N*HMA in DMSO, CD_3_OD and CDCl_3_ (298 K).

DMSO										
δ (*E*)-enol-imine	C(A)	C(B)	C(C)	C(D)	C(E)	C(F)	C(H)	C(I)	C(K)	C(L)
	162.26 (170.23)	130.09 (139.23/136.99) [138.11]	115.85 (119.90)	111.02 125.20 (112.31)	121.47 (131.47/123.97) [127.72]	125.20 (136.19)	56.03 (58.78)	27.09 (29.92)	151.22 (159.85)	148.51 (155.36)
	C(M)	C(N)	C(O)	C(P)						
	128.00 (136.70)	156.62 (167.60)	134.39 (139.67)	197.36 (208.74)						
CD_3_OD										
δ (*E*)-enol-imine	C(A)	C(B)	C(C)	C(D)	C(E)	C(F)	C(H)	C(I)	C(K)	C(L)
	163.06 (170.16)	130.03 (139.17/133.99) [138.08]	115.30 (119.89)	113.26 103.41 (112.30)	127.08 (131.44/123.97) [127.69]	125.44 (136.15)	51.34 (58.75)	25.47 (29.88)	153.49 (159.83)	147.82 (155.34)
	C(M)	C(N)	C(O)	C(P)						
	127.08 (136.69)	156.78 (167.58)	134.39 (139.67)	198.83 (208.61)						
δ (*E*)-keto-amine	C(A’’)	C(B’’)	C(C’’)	C(D’’)	C(E’’)	C(F’’)	C(H’’)	C(I’’)	C(K’’)	C(L’’)
	103.80 ^b^ (146.18)	131.17 (139.88/137.40) [138.64]	129.65 (136.45)	119.73 (133.32)	113.09 (123.96/117.14) [120.33]	114.32 (133.33)	55.52 (61.66)	24.89 (29.81)	198.19 (188.06)	154.37 (160.07)
	C(M’’)	C(N’’)	C(O’’)	C(P’’)						
	110.10 (117.34)	147.82 (150.82)	146.50 (138.99)	198.30 (205.75)						

^a^ δ values, in ppm, relative to Me_4_Si, using the signals of solvents, DMSO and CD_3_OD (δ_C_ = 40 and 48 ppm, respectively) as internal references. ^b^ The experimental and the calculated chemical shifts are not in accordance. According to HSQC, COSY, and HMBC two-dimensional spectra, the proton observed at 5.25 ppm (A’’), which is directly bonded to the carbon observed at 103.80 ppm, has long-range couplings with the E’’ and F’’ protons and has the carbons labeled M’’, D’’ and H’’ (CH_3_) as vicinal carbon atoms. Based on these data, we have assigned the signal observed at 103.80 ppm to the carbon atom A’’. This assignment is further supported by the fact that the NH proton (J’’) is also vicinal to the A’’ carbon atom.

## Data Availability

The original contributions presented in this study are included in the article/Supplementary Material. Further inquiries can be directed to the corresponding authors.

## Supplementary Materials

The following supporting information can be downloaded at: https://www.mdpi.com/article/10.3390/molecules30030745/s1. Figures S1–S4: with the DFT(B3LYP)/6-311++G(d,p) optimized structures of the conformers of the (*E*)- and (*Z*)-isomers of the enol-imine and keto-amine tautomers of A*N*HMA; Figure S5: with the FTIR spectra of A*N*HMA in DMSO-d_6_ and in KBr pellet; Figure S6: with estimation of the relative energies of specific interactions (H-bond or H-bond like) of (*E*)-enol-imine and (*Z*)-keto-amine forms in DMSO, chloroform and methanol, relative to gas phase; Figure S7: Bidimensional (H-H) NMR spectrum (COSY) of A*N*HMA in DMSO-d_6_; Figures S8–S18, with the ^1^H and ^13^C NMR bidimensional data; Table S1: with the B3LYP/6-311++G(d,p) calculated dipole moments and energies for the different conformers of the tautomers of A*N*HMA; Tables S2–S5: with the results of TD-DFT(B3LYP)/6-311++G(d,p) for the different conformers of the tautomers of A*N*HMA.
