# Supplementary material for: Reversal in Solvatochromism, enol-imine/keto-amine Tautomerism and (E)-(Z) Photoisomerizations in a Benzylidene Aniline Schiff Base Derivative in Different Solvents
Source: Molecules. 2025 Feb 6;30(3):745. doi: 10.3390/molecules30030745 (PMC11821250; doi:10.3390/molecules30030745)
Supplement: Supplementary file 1 [file molecules-30-00745-s001.zip › molecules-3144081-supplementary.pdf]

Supporting Information for:

Reversal in Solvatochromism, enol-imine/keto-amine Tautomerism and (*E*)-(*Z*) Photoisomerizations in a Benzylidene Aniline Schiff Base Derivative in Different Solvents

İsa Sıdır [1,2,*], Yadigar Gülseven Sıdır [1,2], Halil Berber [3], Maria L. Ramos [2], Licínia L. G. Justino [2] and Rui Fausto [2,4,*]

1 Department of Physics, Faculty of Sciences and Letters, Bitlis Eren University, Bitlis 13000, Türkiye; ygsidir@beu.edu.tr
2 CQC–IMS, Department of Chemistry, University of Coimbra, 3004-535 Coimbra, Portugal; mlramos@ci.uc.pt (M.L.R.); liciniaj@ci.uc.pt (L.L.G.J.)
3 Department of Chemistry, Faculty of Sciences, Eskişehir Technical University, 26470 Eskişehir, Türkiye; hlberber@eskisehir.edu.tr
4 ERA-Chair Spectroscopy@IKU, Faculty of Sciences and Letters, Department of Physics, Istanbul Kultur University, Ataköy Campus, Bakirköy, Istanbul 34156, Türkiye
* Correspondence: isidir@beu.edu.tr (İ.S.); rfausto@ci.uc.pt (R.F.)

Index:

Page

Figure S1. DFT(B3LYP)/6-311++G(d,p) optimized structures of the conformers of the (*E*)-enol-imine form of A*N*HMA 3

Figure S2. DFT(B3LYP)/6-311++G(d,p) optimized structures of the conformers of the (*Z*)-enol-imine form of A*N*HMA 4

Figure S3. DFT(B3LYP)/6-311++G(d,p) optimized structures of the conformers of the (Z)-keto-amine form of A*N*HMA 5

Figure S4. DFT(B3LYP)/6-311++G(d,p) optimized structures of the conformers of the (*Z*)-keto-amine form of A*N*HMA 6

Figure S5. FTIR spectrum of A*N*HMA in a KBr pellet 7

Figure S6. Estimation of the relative energies of specific interactions (H-bond or H-bond like) of (*E*)-enol-imine and (*Z*)-keto-amine forms in DMSO, chloroform and methanol, relative to gas phase 8

Figure S7. Bidimensional (H-H) NMR spectrum (COSY) of A*N*HMA in DMSO-d_6 9

Figure S8. Bidimensional (H-H) NMR spectrum (NOESY) of A*N*HMA in DMSO-d_6 9

Figure S9. Bidimensional (C-H) NMR spectrum (HSQC) of A*N*HMA in DMSO-d_6 10

Figure S10. Bidimensional (C-H) NMR spectrum (HMBC) of A*N*HMA in DMSO-d_6 10

Figure S11. Bidimensional (H-H) NMR spectrum (COSY) of A*N*HMA in deuterated methanol 11

Figure S12. Bidimensional (H-H) NMR spectrum (NOESY) of A*N*HMA in deuterated methanol 11

Figure S13. Bidimensional (C-H) NMR spectrum (HMQC) of A*N*HMA in deuterated methanol 12

Figure S14. Bidimensional (C-H) NMR spectrum (HMBC) of A*N*HMA in deuterated methanol 12

Figure S15. Bidimensional (H-H) NMR spectrum (COSY) of A*N*HMA in $CDCl_3$ immediately after the dissolution 13

Figure S16. Bidimensional (H-H) NMR spectrum (NOESY) of A*N*HMA in $CDCl_3$ immediately after the dissolution 13

Figure S17. Bidimensional (H-H) NMR spectrum (COSY) of A*N*HMA in $CDCl_3$, 36 h after the dissolution 14

Figure S18. Bidimensional (H-H) NMR spectrum (NOESY) of A*N*HMA in $CDCl_3$, 36 h after the dissolution 14

Table S1. B3LYP/6-311++G(d,p) calculated dipole moments and energies for the different conformers of the tautomers of A*N*HMA 15

Table S2. Results of TD-DFT(B3LYP)/6-311++G(d,p) calculations on the (*E*)-enol-imine conformers 16

Table S3. Results of TD-DFT(B3LYP)/6-311++G(d,p) calculations on the (*Z*)-enol-imine conformers 17

Table S4. Results of TD-DFT(B3LYP)/6-311++G(d,p) calculations on the (*Z*)-keto-amine conformers 18

Table S5. Results of TD-DFT(B3LYP)/6-311++G(d,p) calculations on the (*E*)-keto-amine conformers 19

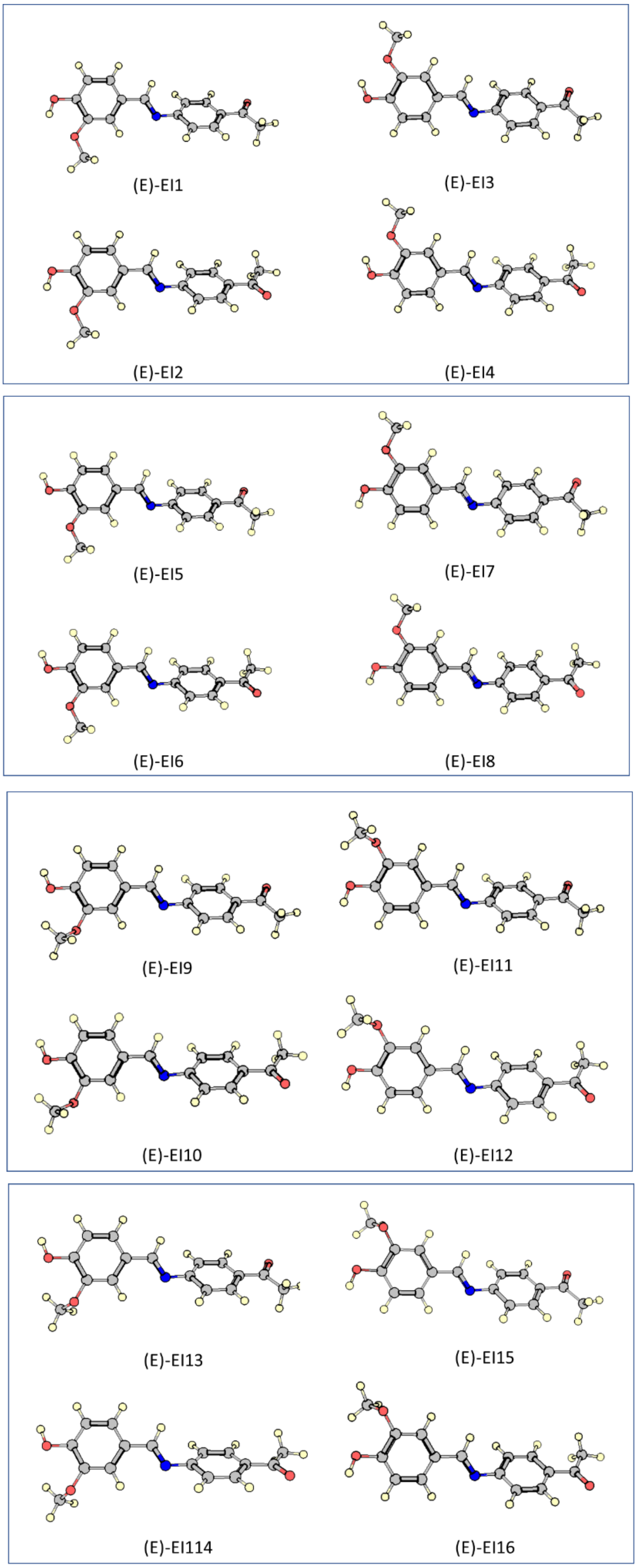

Figure S1. DFT(B3LYP)/6-311++G(d,p) optimized structures of the conformers of the (*E*)-enol-imine form of A*N*HMA.

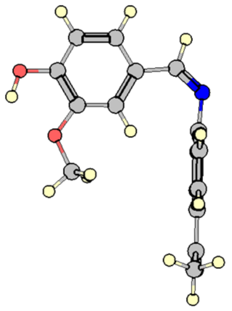

(Z)-EI1

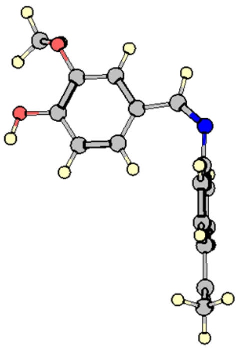

(Z)-EI5

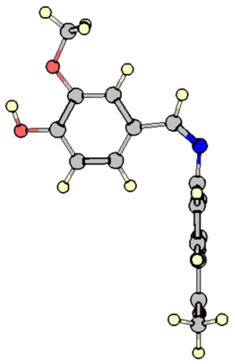

(Z)-EI2

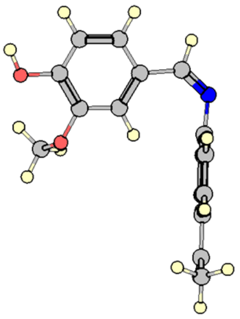

(Z)-EI6

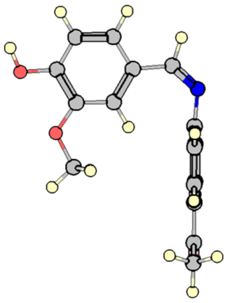

(Z)-EI3

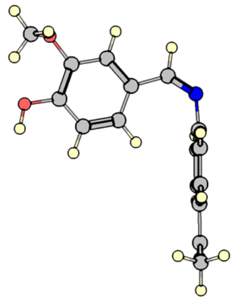

(Z)-EI7

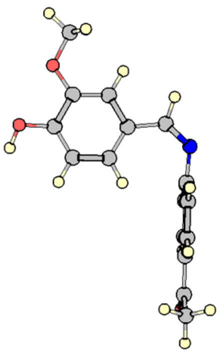

(Z)-EI4

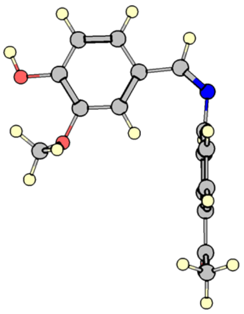

(Z)-EI8

Figure S2. DFT(B3LYP)/6-311++G(d,p) optimized structures of the conformers of the (*Z*)-enol-imine form of A*N*HMA.

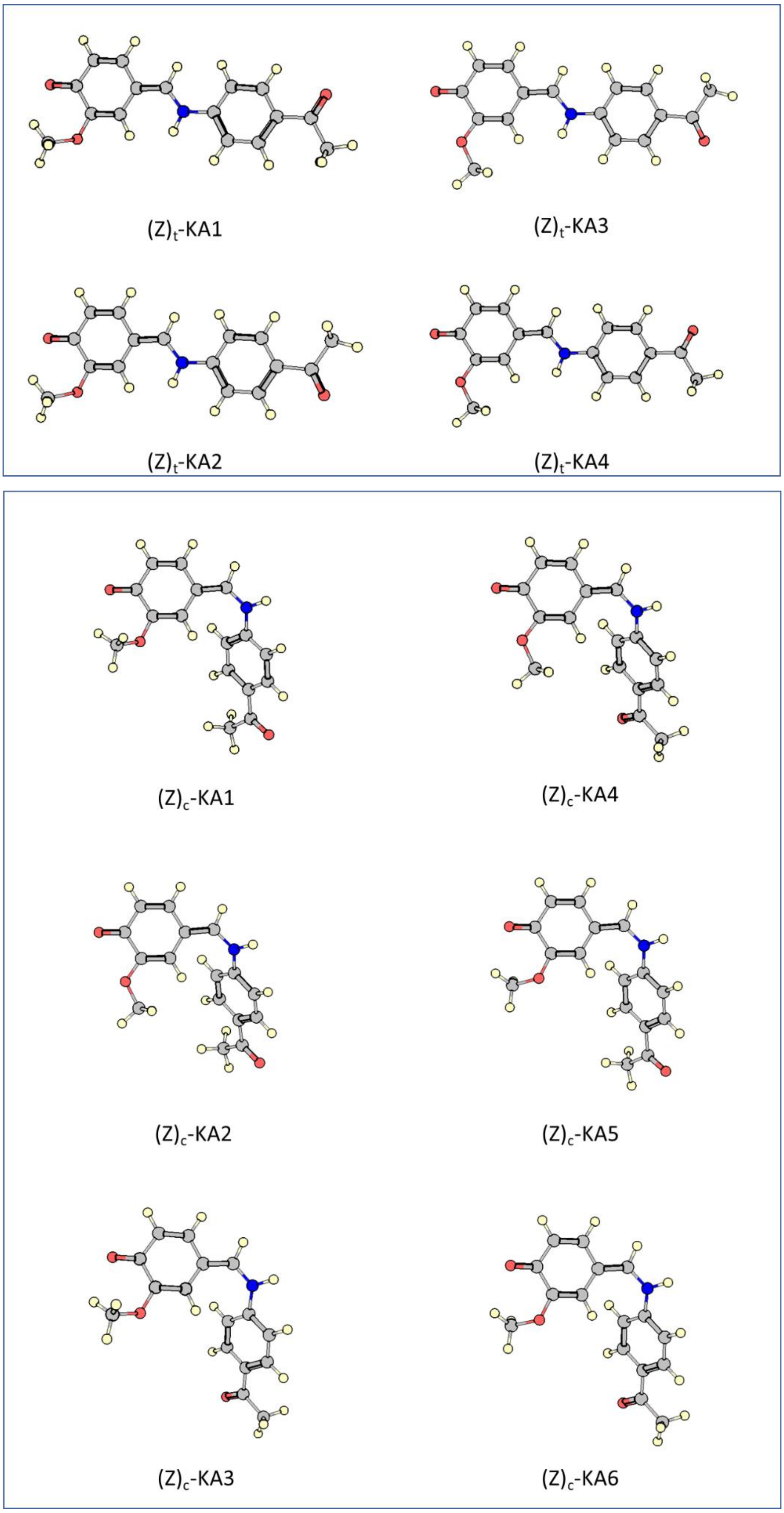

Figure S3. DFT(B3LYP)/6-311++G(d,p) optimized structures of the conformers of the (*Z*)-keto-amine form of A*N*HMA.

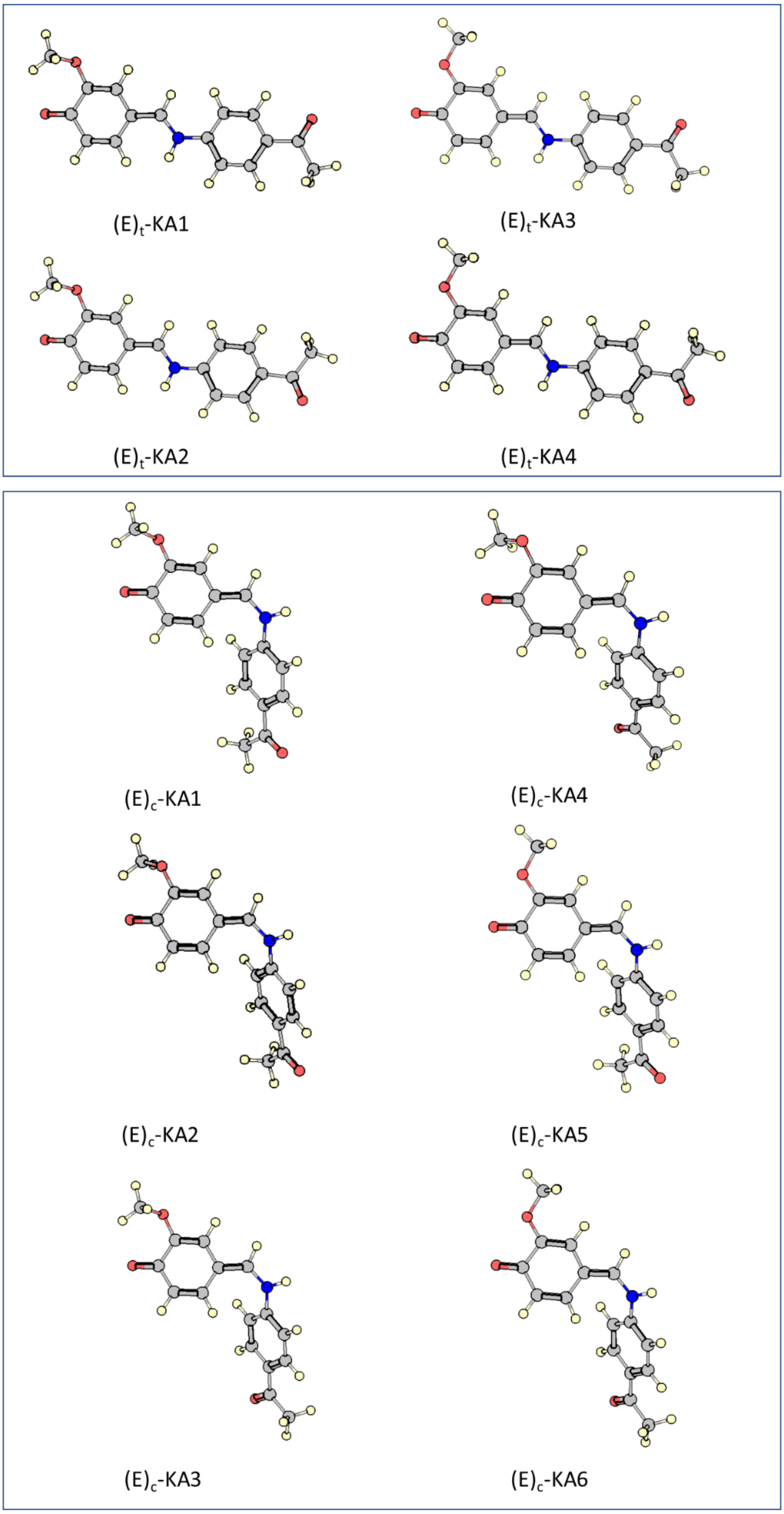

Figure S4. DFT(B3LYP)/6-311++G(d,p) optimized structures of the conformers of the (*Z*)-keto-amine form of A*N*HMA.

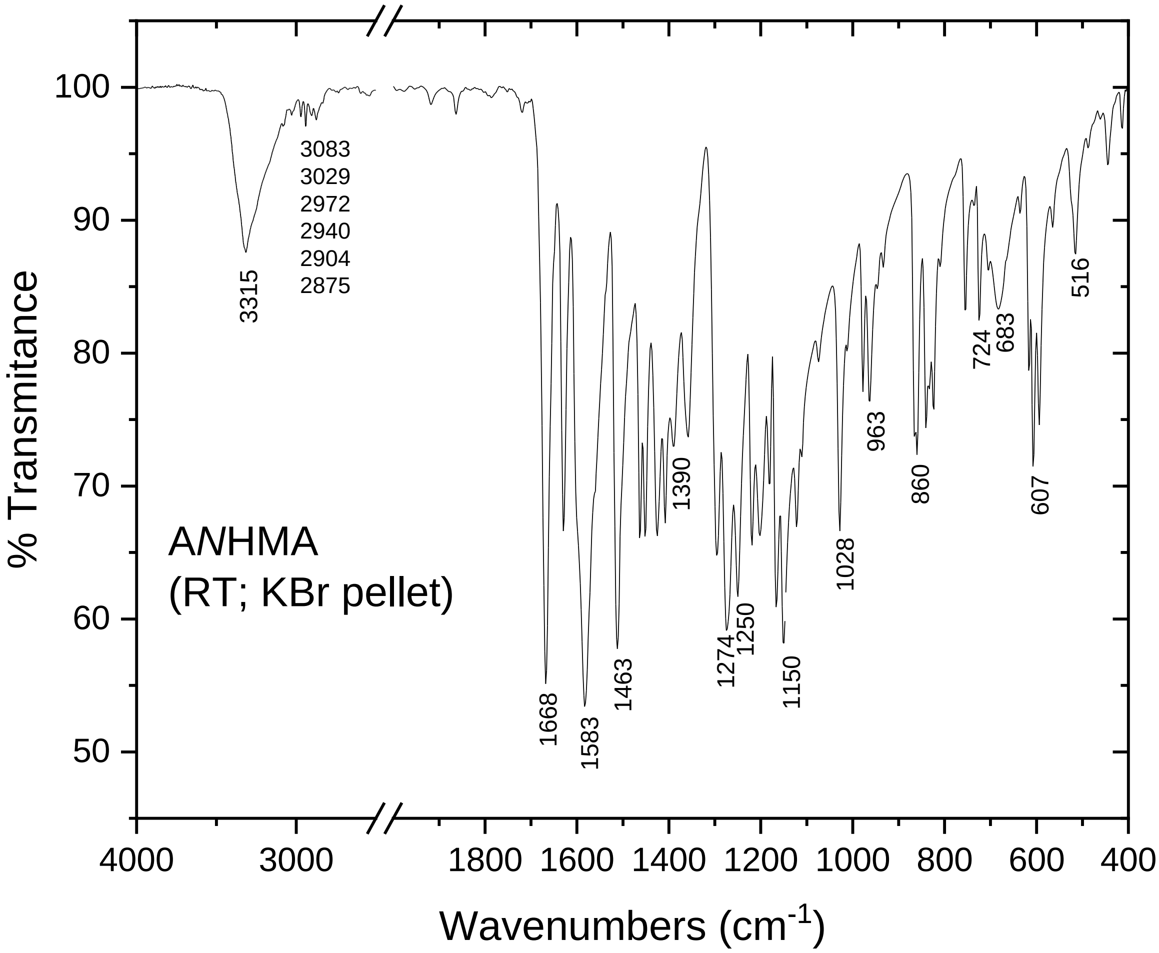

Figure S5. Room temperature FTIR spectrum of A*N*HMA in a KBr pellet.

Interaction	Energy range	Average
N-H…O	10–40	25
N-H…Cl	5–25	15
O-H…O	20–50	35
O-H…N	15–40	27.5
O-H…Cl	5–25	15
C-H…O	1–5	3
C-H…N	1–5	3

Energies in kJ/mol

$\sim\Delta E_{(sol\text{-}gas)}$

	Enol-imine			Keto-amine		
	$CHCl_3$	DMSO	CH_3OH	$CHCl_3$	DMSO	CH_3OH
					-25	-25
				-15		
	35	0	-130			-130
			-27.5			-27.5
	-15					
	-9	-9	3	-9	-9	3
	-3	-3	9	-3	-3	
Total	8	-12	-145.5	-27	-37	-179.5
				-35	-25	-34 $\sim\Delta E_{(keto\text{-}enol)}$

Energies in kJ/mol

Keto-amine

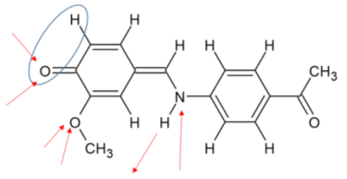

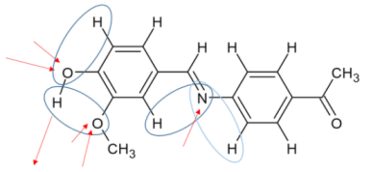

Enol-imine

Figure S6. Energy ranges and average values of different types of H-bond (or H-bond like) interactions and a very approximate model for estimation of the stabilization of the keto-amine tautomer of A*N*HMA relative to the (*E*)-enol-imine tautomer in solution, compared to gas phase. The different interactions in the two forms are marked in the corresponding molecular graphs, those in red corresponding to interactions formed in solution that are absent for the isolated molecule, and those in blue the opposite situation. Interactions that are equal in the two tautomers are ignored. The table at the middle of the figure shows the resulting stabilizing/destabilizing energies for each type of interaction when passing from gas phase to solution in chloroform, DMSO or methanol.

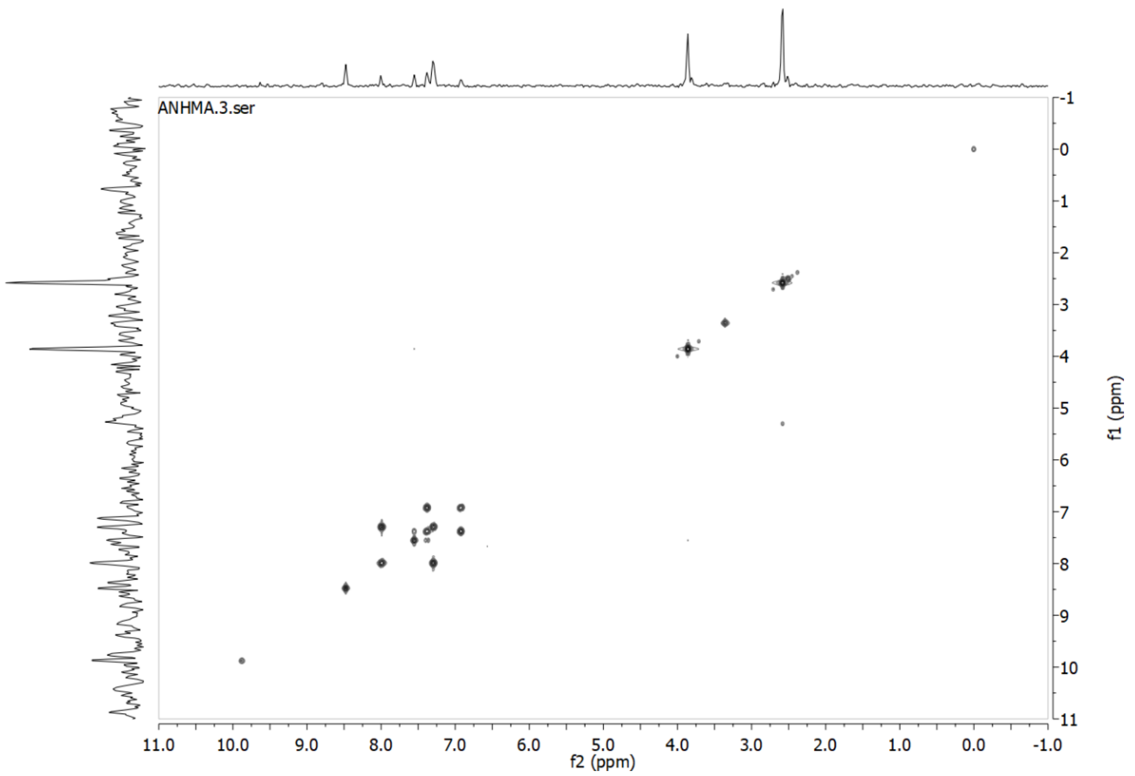

Figure S7. Bidimensional (H-H) NMR spectrum (COSY) of A*N*HMA in DMSO-d$_6$.

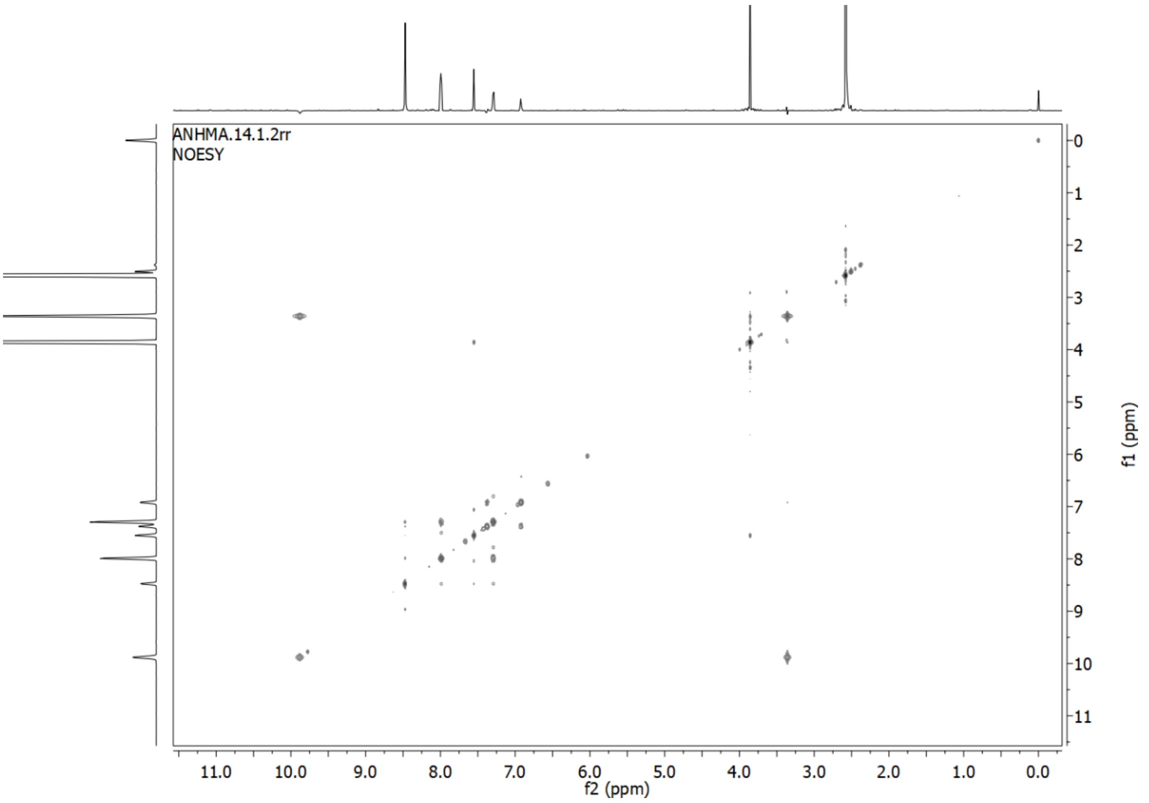

Figure S8. Bidimensional (H-H) NMR spectrum (NOESY) of A*N*HMA in DMSO-d$_6$.

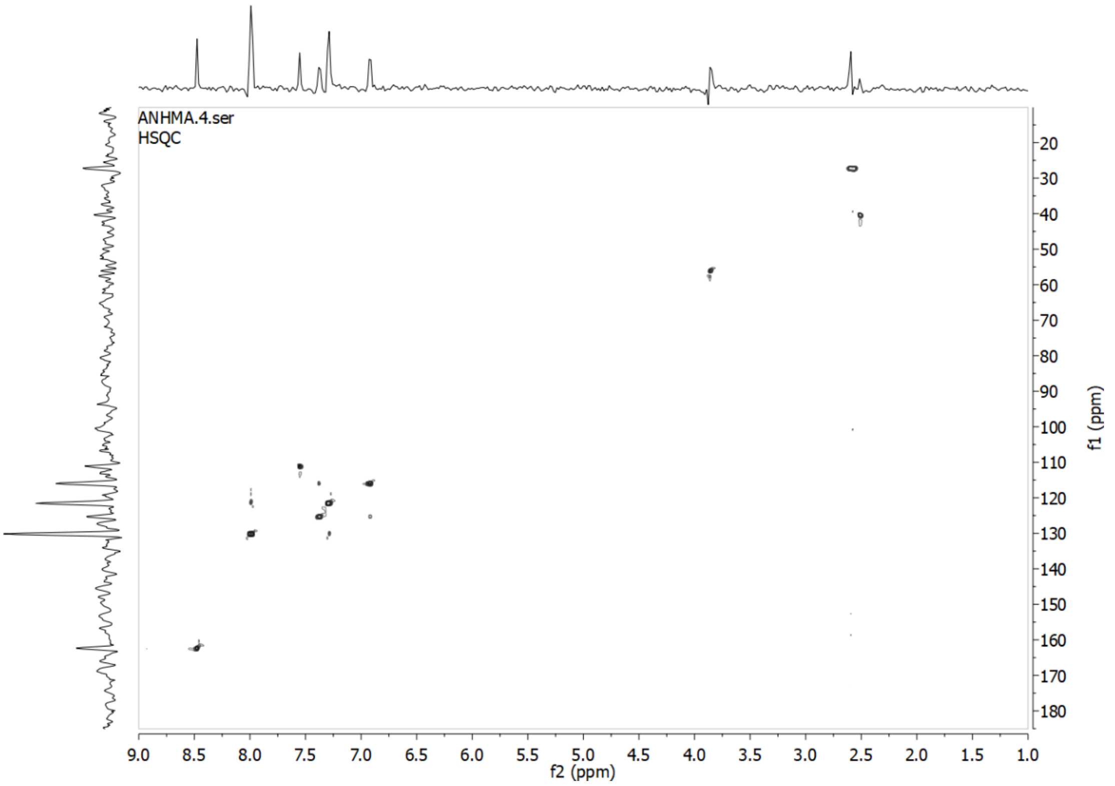

Figure S9. Bidimensional (C-H) NMR spectrum (HSQC) of A*N*HMA in DMSO-d_6.

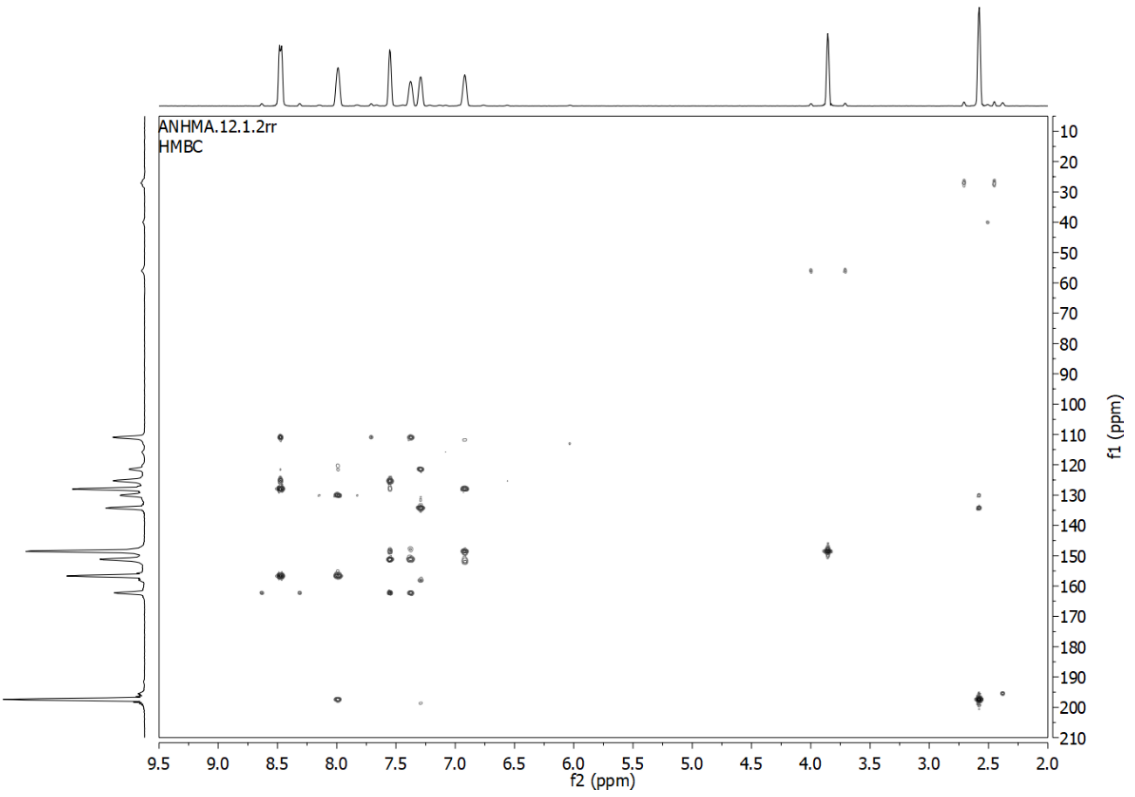

Figure S10. Bidimensional (C-H) NMR spectrum (HMBC) of A*N*HMA in DMSO-d_6.

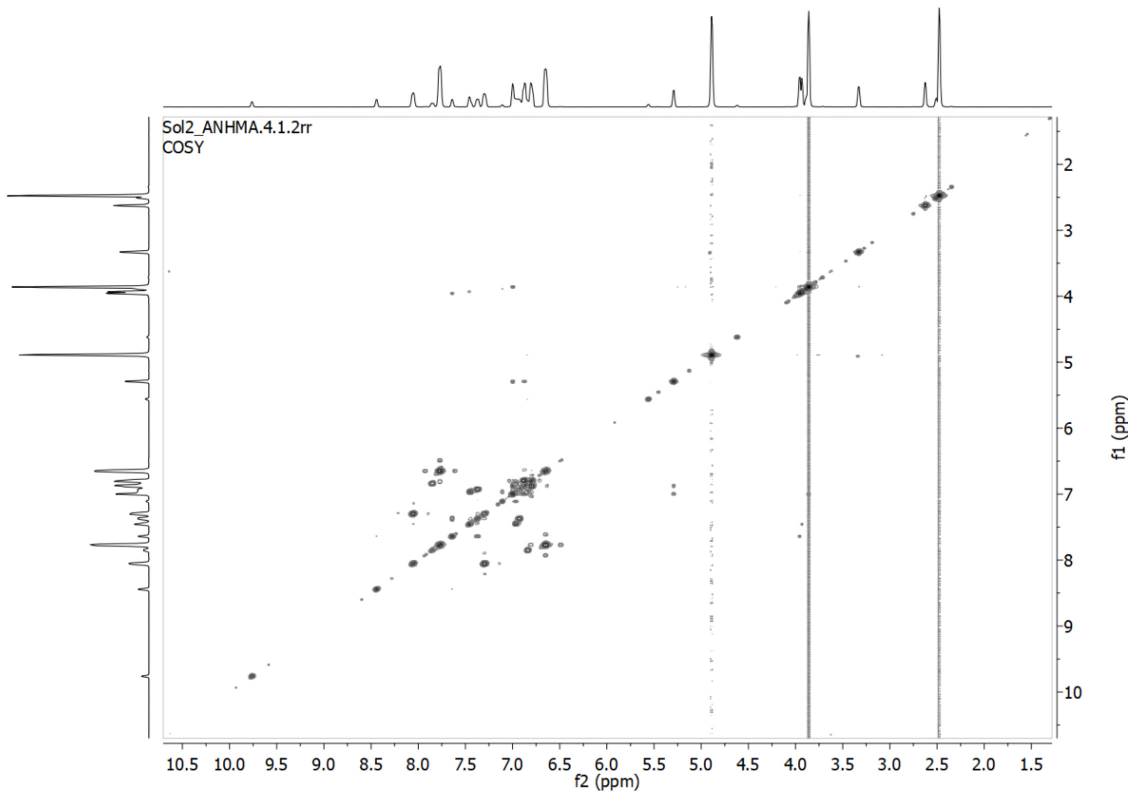

Figure S11. Bidimensional (H-H) NMR spectrum (COSY) of A*N*HMA in deuterated methanol.

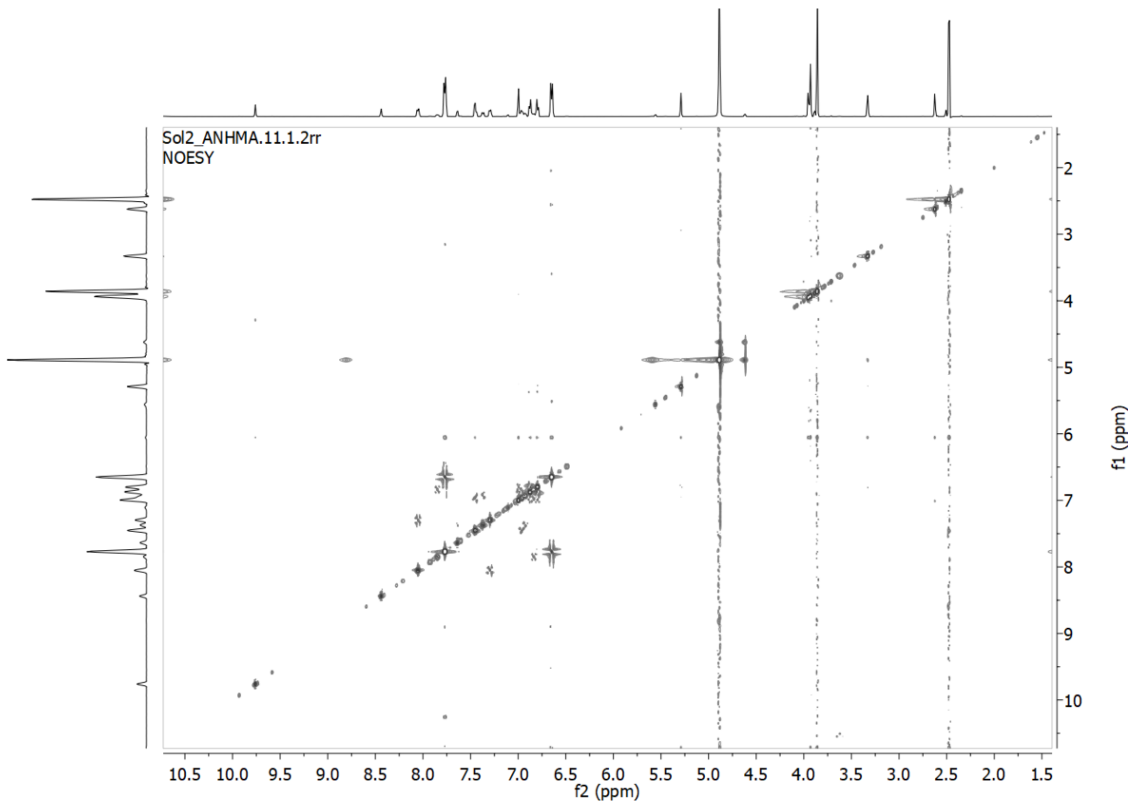

Figure S12. Bidimensional (H-H) NMR spectrum (NOESY) of A*N*HMA in deuterated methanol.

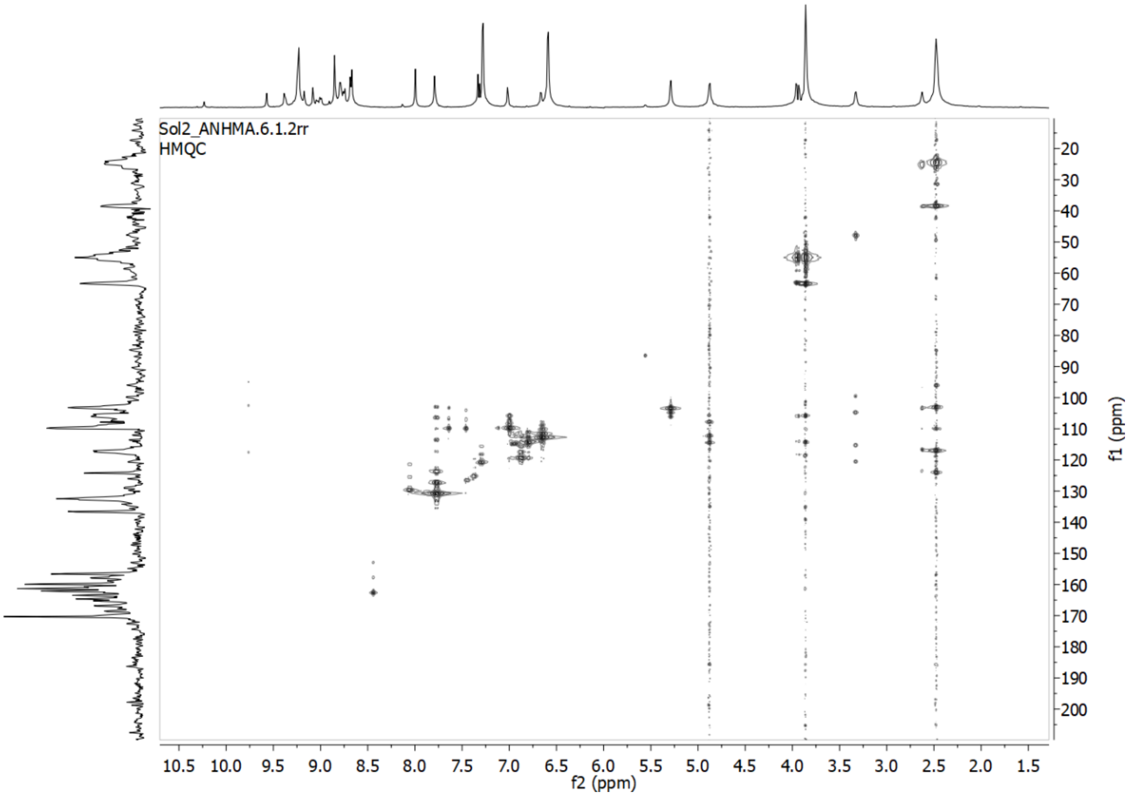

.

Figure S13. Bidimensional (C-H) NMR spectrum (HMQC) of A*N*HMA in deuterated methanol.

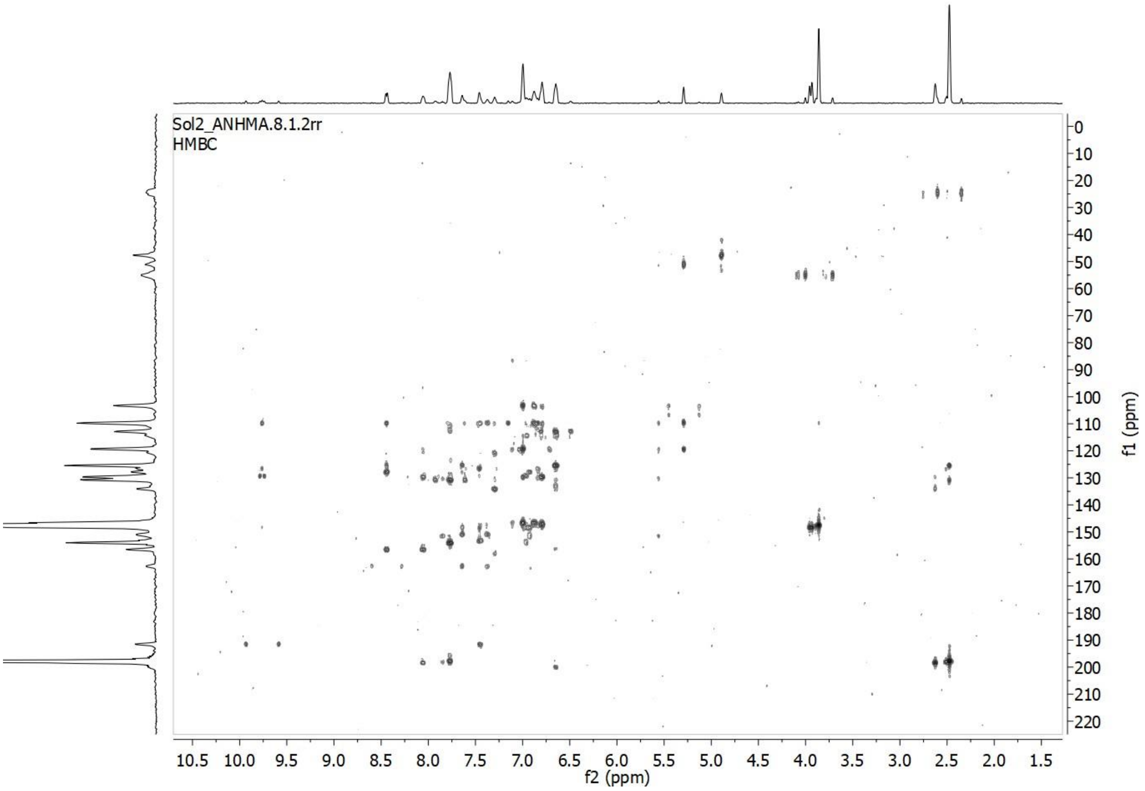

Figure S14. Bidimensional (C-H) NMR spectrum (HMBC) of A*N*HMA in deuterated methanol.

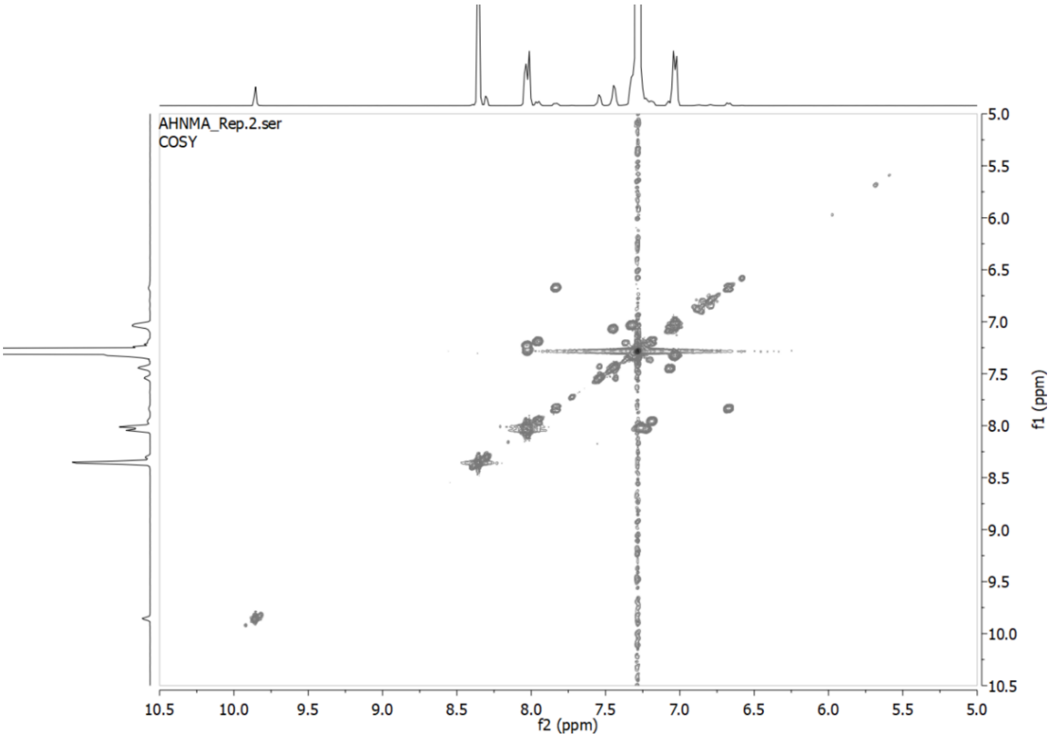

Figure S15. Bidimensional (H-H) NMR spectrum (COSY) of A*N*HMA in $CDCl_3$ immediately after the dissolution.

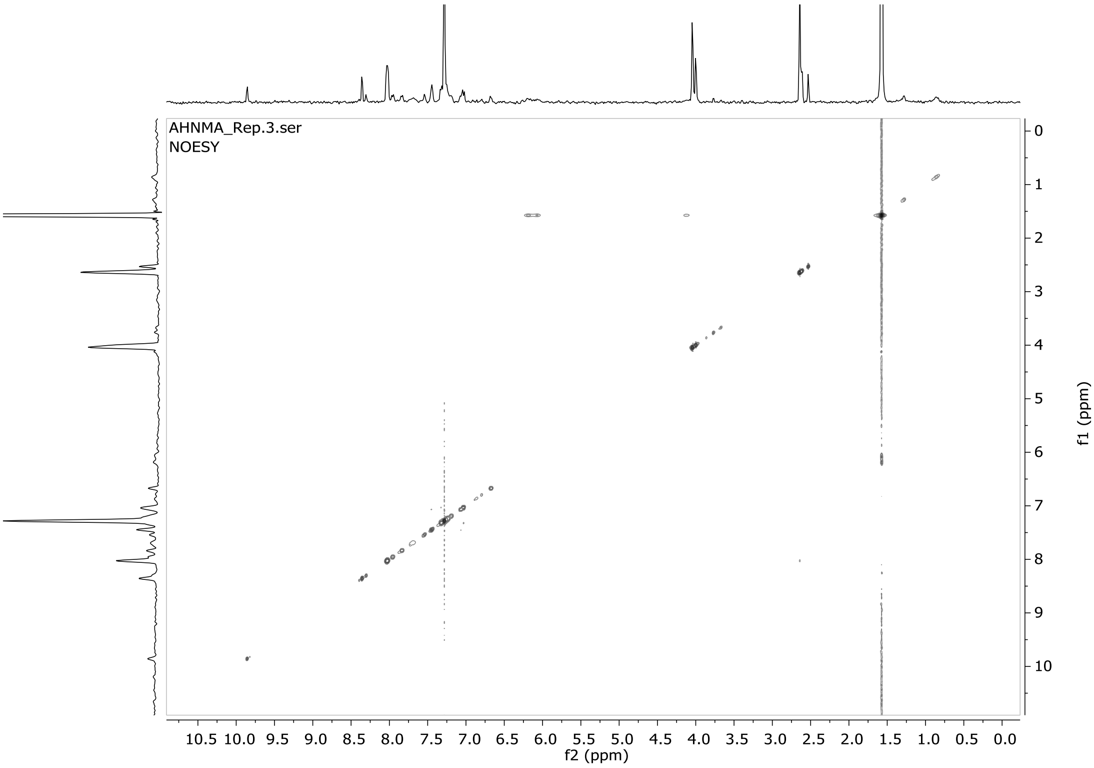

Figure S16. Bidimensional (H-H) NMR spectrum (NOESY) of A*N*HMA in $CDCl_3$ immediately after the dissolution.

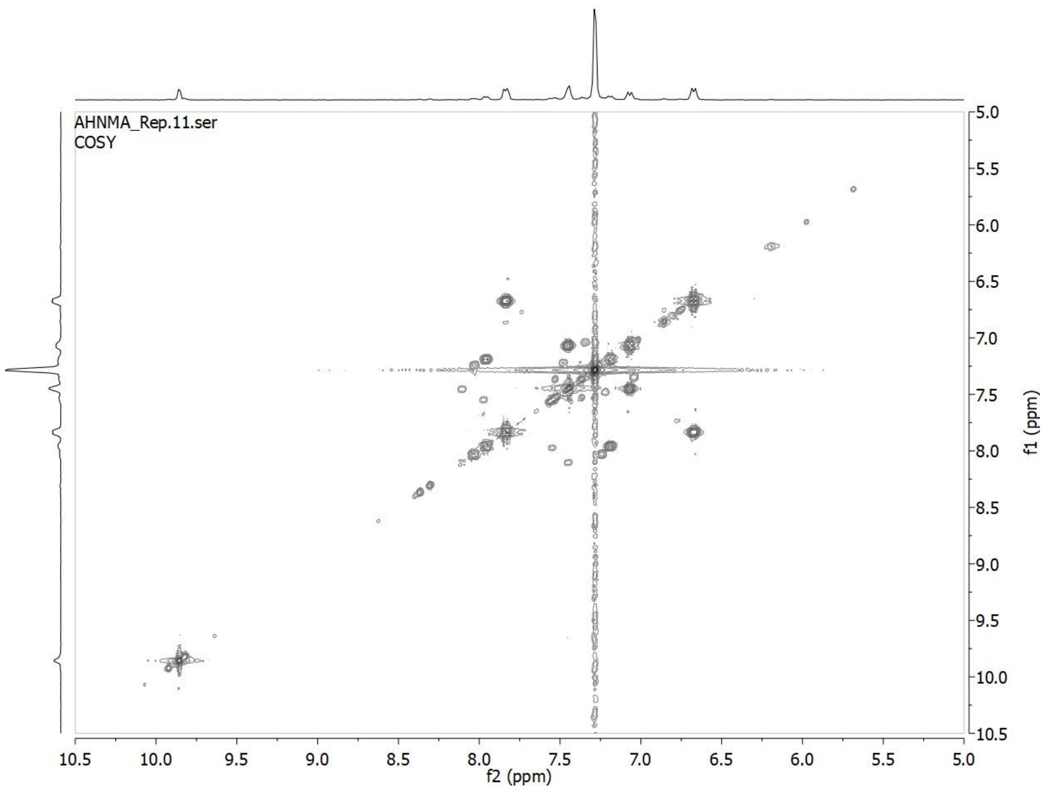

Figure S17. Bidimensional (H-H) NMR spectrum (COSY) of A*N*HMA in $CDCl_3$, 36 h after the dissolution.

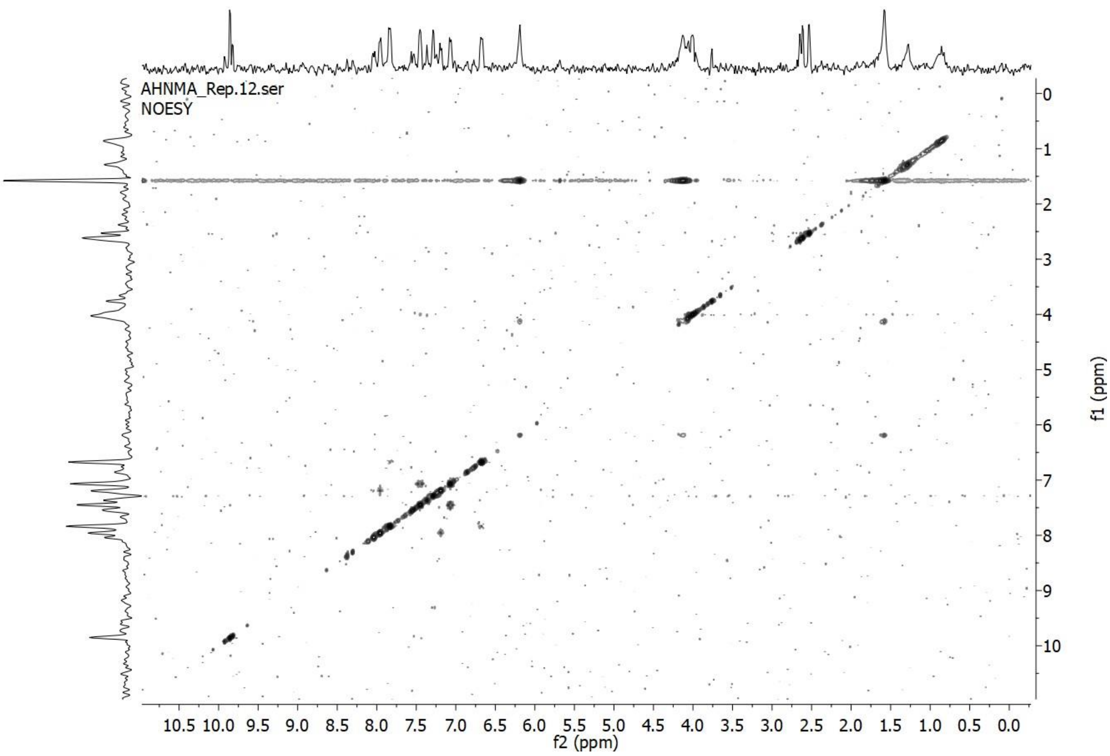

Figure S18. Bidimensional (H-H) NMR spectrum (NOESY) of A*N*HMA in $CDCl_3$, 36 h after the dissolution.

Table S1. B3LYP/6-311++G(d,p) calculated dipole moments (μ), relative electronic energies (ΔE_{el}), zero-point corrected electronic energies (ΔE_0), and standard Gibbs energies ($\Delta G^o_{298.15}$) for the (*E*) and (*Z*) isomers of the enol-imine and keto-amine tautomers of A*N*HMA.[a]

	μ	ΔE_{el}	ΔE_0	$\Delta G^o_{298.15}$	ΔE_{el}	ΔE_0	$\Delta G^o_{298.15}$	ΔE_{el}	ΔE_0	$\Delta G^o_{298.15}$
		Ref. (*E*)-EI1								
(*E*)-Enol-imine										
(*E*)-EI1	4.9	0.0	0.0	0.0						
(*E*)-EI2	4.1	0.4	0.4	0.3						
(*E*)-EI3	4.6	5.2	5.2	5.5						
(*E*)-EI4	7.0	6.0	5.8	6.0						
(*E*)-EI5	4.1	20.9	19.9	19.9						
(*E*)-EI6	5.4	21.4	20.3	20.1						
(*E*)-EI7	4.1	25.0	23.7	23.9						
(*E*)-EI8	5.2	25.5	24.0	24.1						
(*E*)-EI9	5.8	29.6	27.4	26.1						
(*E*)-EI10	6.9	30.2	27.9	26.6						
(*E*)-EI11	5.9	29.5	27.5	27.0						
(*E*)-EI12	5.3	29.6	27.5	26.9						
(*E*)-EI13	5.0	29.5	27.3	26.2						
(*E*)-EI14	7.5	30.3	27.9	26.5						
(*E*)-EI15	5.2	29.5	27.5	26.8						
(*E*)-EI16	5.9	29.9	27.8	27.0						
(*Z*)-Enol-imine										
					Ref. (*Z*)-EI1					
(*Z*)-EI1	3.3	23.6	24.4	22.4	0.0	0.0	0.0			
(*Z*)-EI2	7.3	28.4	29.0	26.6	4.8	4.6	4.2			
(*Z*)-EI3	5.2	44.9	44.4	42.0	21.4	20.0	19.6			
(*Z*)-EI4	5.0	47.4	47.1	44.2	23.9	22.6	21.8			
(*Z*)-EI5	4.3	52.3	51.3	46.7	28.7	26.9	24.3			
(*Z*)-EI6	6.4	52.8	51.4	47.7	29.2	27.0	25.3			
(*Z*)-EI7	5.6	52.5	51.4	47.7	28.9	27.0	25.3			
(*Z*)-EI8	7.4	53.8	52.6	50.2	30.2	28.2	27.8			
(*Z*)-Keto-amine										
C-N *trans*					Ref. $(Z)_t$-KA1					
$(Z)_t$-KA1	4.6	48.5	47.9	42.2	0.0	0.0	0.0			
$(Z)_t$-KA2	4.1	48.4	47.9	42.9	-0.1	0.0	0.7			
$(Z)_t$-KA3	4.6	47.9	48.3	46.0	-0.6	0.4	3.8			
$(Z)_t$-KA4	6.3	48.4	48.6	46.2	-0.2	0.7	4.0			
C-N *cis*								Ref, $(Z)_c$-KA1		
$(Z)_c$-KA1	5.1	73.4	73.6	72.2	24.9	25.4	26.2	0.0	0.0	0.0
$(Z)_c$-KA2	5.6	70.8	71.8	72.6	22.3	23.9	26.7	-2.6	-1.8	0.5
$(Z)_c$-KA2	8.7	75.7	75.6	73.2	27.2	27.4	27.2	2.3	2.0	1.0
$(Z)_c$-KA3	9.4	71.8	72.8	73.6	23.3	24.9	27.6	-1.6	-0.8	1.4
$(Z)_c$-KA4	5.2	75.1	75.3	74.0	26.6	27.4	28.0	1.3	1.3	1.8
$(Z)_c$-KA5	8.0	76.1	76.1	74.5	27.6	28.3	28.6	2.3	2.5	2.4
(*E*)-Keto-amine										
C-N *trans*					Ref. $(E)_t$-KA1					
$(E)_t$-KA1	5.5	53.5	52.8	48.6	0.0	0.0	0.0			
$(E)_t$-KA2	4.2	53.2	52.6	49.2	-0.3	-0.1	0.6			
$(E)_t$-KA3	5.1	52.6	52.8	49.6	-0.9	0.1	1.0			
$(E)_t$-KA4	5.5	52.6	52.9	50.3	-0.9	0.2	1.7			
C-N *cis*								Ref. $(E)_c$-KA1		
$(E)_c$-KA1	5.1	77.6	77.7	76.6	24.1	24.8	27.7	0.0	0.0	0.0
$(E)_c$-KA2	5.4	77.9	78.1	77.0	24.4	25.2	27.1	0.3	0.4	0.4
$(E)_c$-KA3	9.0	79.2	79.1	77.6	25.7	26.2	28.7	1.6	1.4	1.0
$(E)_c$-KA4	8.3	79.2	79.1	77.7	25.7	26.2	28.9	1.6	1.4	1.1
$(E)_c$-KA5	6.6	76.8	77.7	78.0	23.3	24.8	29.1	-0.8	0.0	1.4
$(E)_c$-KA6	9.7	78.3	78.9	78.8	24.8	26.0	29.9	0.7	1.2	2.2

[a] Energy values are in kJ mol^{-1}. Dipole moment in Debye.

Table S2. Results of TD-DFT(B3LYP)/6-311++G(d,p) calculations on the (*E*)-enol-imine conformers (vertical excitation wavelengths, *λ* (nm), oscillator strengths, *f*, and main contributions to the excited states).

	State	*λ*	*f*	Major contributions
(*E*)-Enol-imine				
(*E*)-EI1	S_1	348.0	0.7956	HOMO→LUMO (66%)
	S_2	339.4	0.0203	HOMO-2→LUMO (59%)
	S_3	314.4	0.0245	HOMO-1→LUMO (59%)
(*E*)-EI2	S_1	347.2	0.8065	HOMO→LUMO (67%)
	S_2	339.2	0.0028	HOMO-2→LUMO (60%)
	S_3	314.1	0.0269	HOMO-1→LUMO (60%)
(*E*)-EI3	S_1	350.5	0.7673	HOMO→LUMO (67%)
	S_2	339.4	0.0088	HOMO-2→LUMO (59%)
	S_3	318.6	0.0067	HOMO-1→LUMO (60%)
(*E*)-EI4	S_1	349.0	0.7630	HOMO→LUMO (67%)
	S_2	338.9	0.0020	HOMO-2→LUMO 60%)
	S_3	317.8	0.0098	HOMO-1→LUMO (61%)
(*E*)-EI5	S_1	349.4	0.7624	HOMO→LUMO (67%)
	S_2	339.4	0.0101	HOMO-2→LUMO (59%)
	S_3	320.0	0.0021	HOMO-1→LUMO (62%)
(*E*)-EI6	S_1	348.4	0.7641	HOMO→LUMO (67%)
	S_2	339.0	0.0018	HOMO-2→LUMO (61%)
	S_3	319.8	0.0020	HOMO-1→LUMO (63%)
(*E*)-EI7	S_1	353.7	0.7000	HOMO→LUMO (68%)
	S_2	339.6	0.0018	HOMO-2→LUMO 60%)
	S_3	323.5	0.0174	HOMO-1→LUMO (62%)
(*E*)-EI8	S_1	352.3	0.6964	HOMO→LUMO (68%)
	S_2	339.0	0.0022	HOMO-2→LUMO (61%)
	S_3	322.9	0.0131	HOMO-1→LUMO (63%)
(*E*)-EI9	S_1	344.7	0.6069	HOMO→LUMO (61%)
	S_2	339.7	0.1024	HOMO-2→LUMO (55%)
	S_3	306.2	0.1566	HOMO-1→LUMO (56%)
(*E*)-EI10	S_1	343.1	0.6884	HOMO→LUMO (65%)
	S_2	339.7	0.0002	HOMO-2→LUMO (58%)
	S_3	305.2	0.1653	HOMO-1→LUMO (57%)
(*E*)-EI11	S_1	345.0	0.6341	HOMO→LUMO (62%)
	S_2	339.9	0.0871	HOMO-2→LUMO (56%)
	S_3	310.8	0.0731	HOMO-1→LUMO (58%)
(*E*)-EI12	S_1	343.7	0.7055	HOMO→LUMO (66%)
	S_2	340.0	0.0027	HOMO-2→LUMO (59%)
	S_3	309.9	0.0804	HOMO-1→LUMO (59%)
(*E*)-EI13	S_1	344.5	0.5877	HOMO→LUMO (60%)
	S_2	339.2	0.1154	HOMO-2→LUMO (55%)
	S_3	307.6	0.1668	HOMO-1→LUMO (57%)
(*E*)-EI14	S_1	342.5	0.6824	HOMO→LUMO (65%)
	S_2	339.5	0.0003	HOMO-2→LUMO (59%)
	S_3	306.6	0.1759	HOMO-1→LUMO (58%)
(*E*)-EI15	S_1	344.7	0.6239	HOMO→LUMO (61%)
	S_2	339.7	0.0994	HOMO-2→LUMO (56%)
	S_3	312.2	0.0708	HOMO-1→LUMO (59%)
(*E*)-EI16	S_1	343.3	0.7052	HOMO→LUMO (65%)
	S_2	339.9	0.0032	HOMO-2→LUMO (60%)
	S_3	311.4	0.0840	HOMO-1→LUMO (60%)

Table S3. Results of TD-DFT(B3LYP)/6-311++G(d,p) calculations on the (*Z*)-enol-imine forms (vertical excitation wavelengths, *λ* (nm), oscillator strengths, *f*, and main contributions to the excited states).

	State	*λ*	*f*	Major contributions
(*Z*)-Enol-imine				
(*Z*)-EI1	S_1	350.4	0.0022	HOMO→LUMO (68%)
	S_2	325.2	0.0007	HOMO-2→LUMO+1 (66%)
	S_3	300.2	0.4756	HOMO-1→LUMO (49%)
(*Z*)-EI2	S_1	356.6	0.0002	HOMO→LUMO (70%)
	S_2	325.2	0.0001	HOMO-2→LUMO+1 (69%)
	S_3	296.8	0.4747	HOMO-1→LUMO (51%)
(*Z*)-EI3	S_1	352.6	0.0008	HOMO→LUMO (69%)
	S_2	325.3	0.0004	HOMO-2→LUMO+1 (68%)
	S_3	302.7	0.4477	HOMO-1→LUMO (56%)
(*Z*)-EI4	S_1	355.3	0.0023	HOMO→LUMO (69%)
	S_2	325.2	0.0002	HOMO-2→LUMO+1 (68%)
	S_3	301.8	0.3057	HOMO-1→LUMO (58%)
(*Z*)-EI5	S_1	357.2	0.0082	HOMO→LUMO (68%)
	S_2	325.5	0.0003	HOMO-2→LUMO+1 (64%)
	S_3	293.4	0.4668	HOMO→LUMO+1 (55%)
(*Z*)-EI6	S_1	360.7	0.0023	HOMO→LUMO (70%)
	S_2	325.2	0.0002	HOMO-2→LUMO+1 (68%)
	S_3	292.0	0.5390	HOMO→LUMO+1 (58%))
(*Z*)-EI7	S_1	356.9	0.0027	HOMO→LUMO (69%)
	S_2	325.4	0.0002	HOMO-2→LUMO+1 (67%)
	S_3	292.9	0.4915	HOMO→LUMO+1 (57%))
(*Z*)-EI8	S_1	361.5	0.0016	HOMO→LUMO (70%)
	S_2	325.4	0.0001	HOMO-2→LUMO+1 (68%)
	S_3	291.9	0.5475	HOMO→LUMO+1 (57%))

Table S4. Results of TD-DFT(B3LYP)/6-311++G(d,p) calculations on the (*Z*)-keto-amine conformers (vertical excitation wavelengths, *λ* (nm), oscillator strengths, *f*, and main contributions to the excited states).

	State	*λ*	*f*	Major contributions
(*Z*)-Keto-amine				
C-N *trans*				
$(Z)_t$-KA1	S_1	431.15	0.1130	HOMO-1→LUMO (61%)
	S_2	398.38	1.0003	HOMO→LUMO (62%)
	S_3	341.99	0.0001	HOMO-3→LUMO (58%)
$(Z)_t$-KA2	S_1	431.81	0.1126	HOMO-1→LUMO (60%)
	S_2	398.73	0.9978	HOMO→LUMO (62%)
	S_3	342.08	0.0001	HOMO-3→LUMO (58%)
$(Z)_t$-KA3	S_1	411.35	0.0000	HOMO-1→LUMO (67%)
	S_2	397.69	1.1161	HOMO→LUMO (69%)
	S_3	339.53	0.0001	HOMO-3→LUMO (58%)
$(Z)_t$-KA4	S_1	410.99	0.0000	HOMO-1→LUMO (67%)
	S_2	397.24	1.1174	HOMO→LUMO (69%)
	S_3	339.56	0.0001	HOMO-3→LUMO (58%)
C-N *cis*				
$(Z)_c$-KA1	S_1	435.16	0.0281	HOMO-1→LUMO (63%)
	S_2	407.14	0.5354	HOMO→LUMO (64%)
	S_3	347.84	0.0423	HOMO-2→LUMO (66%)
$(Z)_c$-KA2	S_1	416.03	0.0352	HOMO-1→LUMO (65%)
	S_2	405.33	0.5241	HOMO→LUMO (66%)
	S_3	343.22	0.0473	HOMO-2→LUMO (65%)
$(Z)_c$-KA3	S_1	435.98	0.0462	HOMO-1→LUMO (61%)
	S_2	409.21	0.4922	HOMO→LUMO (62%)
	S_3	348.82	0.0487	HOMO-2→LUMO (65%)
$(Z)_c$-KA4	S_1	416.61	0.0306	HOMO-1→LUMO (65%)
	S_2	407.74	0.5116	HOMO→LUMO (66%)
	S_3	342.59	0.0453	HOMO-2→LUMO (60%)
$(Z)_c$-KA5	S_1	438.52	0.1240	HOMO-1→LUMO (54%
	S_2	404.94	0.4170	HOMO→LUMO (53%)
	S_3	351.06	0.0623	HOMO-2→LUMO (67%)
$(Z)_c$-KA6	S_1	440.14	0.1150	HOMO-1→LUMO (54%)
	S_2	406.40	0.4007	HOMO→LUMO (53%)
	S_3	350.44	0.0611	HOMO-2→LUMO (66%)

Table S5. Results of TD-DFT(B3LYP)/6-311++G(d,p) calculations on the (*E*)-keto-amine conformers (vertical excitation wavelengths, λ (nm), oscillator strengths, *f*, and main contributions to the excited states).

	State	λ	*f*	Major contributions
(*E*)-Keto-amine				
C-N *trans*				
$(E)_t$-KA1	S_1	444.08	0.1408	HOMO-1→LUMO (58%)
	S_2	409.85	0.7648	HOMO→LUMO (58%)
	S_3	342.66	0.0001	HOMO-3→LUMO (58%)
$(E)_t$-KA2	S_1	444.18	0.1434	HOMO-1→LUMO (58%)
	S_2	409.98	0.7588	HOMO→LUMO (58%)
	S_3	342.63	0.0001	HOMO-3→LUMO (58%)
$(E)_t$-KA3	S_1	420.64	0.8798	HOMO→LUMO (69%)
	S_2	418.30	0.0003	HOMO-1→LUMO (68%))
	S_3	344.75	0.0199	HOMO→LUMO+1 (67%)
$(E)_t$-KA4	S_1	420.40	0.8813	HOMO→LUMO (69%)
	S_2	418.54	0.0003	HOMO-1→LUMO (68%)
	S_3	343.14	0.0244	HOMO→LUMO+1 (67%)
C-N *cis*				
$(E)_c$-KA1	S_1	451.24	0.1331	HOMO-1→LUMO (50%)
	S_2	417.24	0.3121	HOMO→LUMO (50%)
	S_3	344.27	0.0013	HOMO-3→LUMO (56%)
$(E)_c$-KA2	S_1	447.32	0.1029	HOMO-1→LUMO (55%)
	S_2	423.13	0.3425	HOMO→LUMO (54%)
	S_3	344.25	0.0015	HOMO-3→LUMO (56%)
$(E)_c$-KA3	S_1	451.49	0.1311	HOMO-1→LUMO (50%)
	S_2	417.79	0.3067	HOMO→LUMO (49%)
	S_3	345.97	0.0393	HOMO→LUMO+1 (53%)
$(E)_c$-KA4	S_1	447.45	0.0981	HOMO-1→LUMO (55%)
	S_2	422.95	0.3420	HOMO→LUMO (54%)
	S_3	346.17	0.0403	HOMO→LUMO+1 (53%)
$(E)_c$-KA5	S_1	433.40	0.4243	HOMO→LUMO (67%)
	S_2	420.43	0.0198	HOMO-1→LUMO (66%)
	S_3	343.56	0.0432	HOMO→LUMO+1 (60%)
$(E)_c$-KA6	S_1	432.76	0.4236	HOMO→LUMO (67%)
	S_2	420.99	0.0207	HOMO-1→LUMO 66%)
	S_3	346.09	0.0479	HOMO→LUMO+1 (62%)
